# NET-EN treatment leads to delayed HSV-2 infection, enhanced mucin and T cell functions in the female genital tract when compared to DMPA in a preclinical mouse model

**DOI:** 10.3389/fimmu.2024.1427842

**Published:** 2024-11-06

**Authors:** M. Firoz Mian, Sidney Pa, Nuzhat Rahman, Amy Gillgrass, Charu Kaushic

**Affiliations:** ^1^ McMaster Immunology Research Centre, Department of Medicine, McMaster University, Hamilton, ON, Canada; ^2^ Department of Medicine, McMaster University, Hamilton, ON, Canada

**Keywords:** NET-EN, DMPA, herpes simplex virus type 2, IFN-γ, TNF-α, mucin, vaginal tract

## Abstract

Depot-medroxyprogesterone acetate (DMPA) and Norethisterone Enanthate (NET-EN) are progestin-only injectable contraceptives widely used by women in sub-Sharan Africa, where incidence of HIV-1 and HSV-2 infection remains high. Studies indicate that DMPA usage can increase the risk of HSV-2 infection, but limited data indicate no increased risk with use of NET-EN. We therefore investigated the effects of NET-EN and DMPA on susceptibility to vaginal HSV-2 infection in ovariectomized (OVX) mice and effects on immune responses, particularly in the vaginal tract (VT). OVX mice, when treated with NET-EN and infected intravaginally, had delayed genital pathology, decreased viral shedding, and extended survival compared to DMPA- or untreated OVX mice. CD4+ T cells isolated from VT showed no significant change in frequency with either contraceptive. However, DMPA significantly decreased the total number of VT CD4+ and CD8+ T cells and the number of IFN-γ producing CD4 and CD8 T cells and increased the percentage of CD4 and CD8 T cells producing TNF-α compared to untreated mice. In contrast, NET-EN significantly enhanced percentages of CD8+ T cells compared to DMPA treated mice, and frequencies of IFN-γ+ CD4 and CD8 T cells in the VT compared to untreated mice. Comparative analysis of splenic lymphocytes indicated that DMPA treatment resulted in reduction of CD4+ T cell frequency, but enhanced TNF-α+ CD4 T cells compared to untreated mice. NET-EN enhanced the frequency of CD8 T cells, as well as IFN-γ+ and TNF-α+ CD4, and IFN-γ+ CD8 T cells in the spleen compared to untreated mice. Importantly, we found DMPA treatment that significantly reduced mucin production, whereas NET-EN enhanced expression of cell-associated mucin in VT. High levels of mucin in NET-EN mice were associated with lower levels of HSV-2 virus detected in the vaginal tract. This study provides the first evidence that NET-EN treatment can delay HSV-2 infection compared to DMPA.

## 1 Introduction

Depot-medroxyprogesterone acetate (DMPA) and Norethisterone-Enanthate (NET-EN) are both progestin-only injectable contraceptives widely used in preventing conception and unwanted pregnancy, particularly in sub-Sharan African countries (1, 2). The high use of these injectable hormonal contraceptives (HC) overlaps with high prevalence of HIV-1 and HSV-2. A number of epidemiological and clinical studies indicate that DMPA is associated with higher risk of HIV-1 acquisition and other STI such as HSV-2 (3–8). Meta-analyses data show that DMPA is associated with a 40-50% increased risk of HIV-1 compared to women not taking hormonal contraception (1, 2). DMPA was also recently identified as a risk factor for male-to-female sexual transmission of HSV-2 (9). The World Health Organization recently (2020) reported that 102.9 million (43.9%) reproductive age (15-49 years) women in African countries and 57.7 million (24%) in the Americas are infected with HSV-2 (10). While there are fewer studies on NET-EN and STI risk, no such association has been reported (4, 11, 12). Therefore, better understanding of how DMPA and NET-EN affect susceptibility to HIV-1 and HSV-2 remains an important public health issue to offer better and safer contraceptive options to women.

Several mechanisms linking hormonal contraceptives including DMPA to increased STI susceptibility have been described (12, 13). Both DMPA and NET-EN are injectable progestins, but they have different mechanisms of action. DMPA, but not NET-EN, shows higher binding affinity to glucocorticoid receptor (GR) (12, 14, 15). In murine models of vaginal tract infection, we and others have shown that DMPA increases susceptibility to HIV-1 and HSV-2 infections by decreasing cell-cell adhesion molecules, thinning the epithelial layer, breaching barrier integrity, and increasing HIV target cells and vaginal epithelial permeability (4, 5, 16). A recent study revealed comparable susceptibility and mortality rates in DMPA and NET-EN treated wild type and humanized mice after intravaginal infection with HSV-2 and cell-associated HIV-1 (4). Several clinical and ex vivo studies have documented that DMPA is immunosuppressive and impacts both innate and adaptive immunity, which has not been shown for NET-EN (12, 17–19), although controversies exist among studies due to inconsistent results. As reviewed in Hapgood et al. (13), DMPA, but not NET-EN, was shown to suppress the production of several T cell-derived cytokines and chemokines (20). Another study showed both DMPA and NET-EN increase proinflammatory cytokines in the vaginal fluids (21). A recent study in healthy women using pharmacological concentration of DMPA and NET-EN did not show broad immunosuppression effects but did find DMPA and not NET-EN usage resulted in decreased IFN-γ and TNF-α-producing CD4+ and CD8+ T cells after prolonged exposure (22, 23). Furthermore, MPA at physiological concentrations significantly inhibited the activation of T cells isolated from healthy premenopausal women, conversely, no immunosuppressive effects observed with NET-EN treatment (20). It is believed that some combination of CD4+ T cells and CD8+ T cells is indispensable to protect against HSV-2 infection in the lower female genital tract of women (24, 25). Both clinical and experimental studies documented that mucosal T cells play an important role in controlling HSV-2 infection (26, 27). Assessing genital pathology in HSV-2 infected women revealed that virus clearance is heavily dependent on CD8 T cells that participate in active immune surveillance at the sites of HSV-2 reactivation (24, 28). In addition, studies examining HSV-2 in the female reproductive tract (FRT) showed that 90% of HSV-2 specific CD3+ T cells detected were CD4 T cells (28, 29). Likewise, murine model studies have shown that compared to antibody responses, T helper 1 (Th1) immunity and the production of IFN-γ are critical for protection against HSV-2 (28, 30, 31).

In the female reproductive tract, the epithelium of the cervix and its glands secrete mucins that act as a physical and chemical barrier against pathogens in the uterus and vagina. Mucin glycoproteins are key components of innate immunity. These high molecular weight and highly glycosylated proteins are either located at the cell membrane (MUC1, MUC4, MUC13 and MUC16) or are secreted extracellularly (MUC5AC, MUC5B and MUC6) to form mucus gel that covers the moist epithelium. Mucus hydrates, lubricates and acts as the first line of defense against pathogenic bacteria and viruses (32). Evidence indicate that mucin glycoproteins comprise a large proportion of the female reproductive tract tissue epithelial surface. Of these mucins, Muc1 is persistently expressed throughout the reproductive tract mucosa including uterus, cervix and vagina. It plays important roles in normal reproductive functions and in protection from infections (33). Muc1 has also been shown to be a crucial component of innate immunity playing an important anti-inflammatory role during airway infection caused by bacterial and viral pathogens (34–36). Steroid hormones control Muc1 expression which has been studied in mice where estrogen stimulates the mouse Muc1 gene expression at the apical surface of the reproductive tract mucosal epithelium, progesterone in contrast, antagonizes the stimulatory action of estrogen (37, 38). Mucins have emerged as one of the important components of the immune regulatory arsenal which can suppress immune cell recruitment and cytokine production *in vivo* (39, 40). Purified mucins from human breast milk inhibit HIV-1 infection as assessed in an *in vitro* HIV-1 p24 assay and SARS-CoV2 in an infectivity assay using Vero E6 cells (41, 42). Even though the available evidence indicates that the cervical mucus is regulated by hormones and it can pose a significant barrier to entry of pathogens, the effect of hormonal contraceptives on mucus production needs to be studied since it could affect protection against HSV-2 in FRT.

To address the gaps in the field, we performed a comprehensive study using a murine model, to assess *in vivo* the effects of NET-EN and DMPA on HSV-2 susceptibility and the underlying mechanisms with major focus on T cell immune response and vaginal mucus production. We demonstrate that NET-EN slows down HSV-2 infection, compared to DMPA treatment. We further show that DMPA treatment appeared to significantly decrease the number of T cells in the vagina while selectively enhancing the frequency of TNF-α producing CD4 and CD8 T cells. NET-EN, on the other hand, appeared to significantly enhance the frequency of IFN-γ producing CD4 and CD8 T cells. Among splenic lymphocytes, NET-EN enhances the frequency of CD8 T cells and IFN-γ producing CD4 and CD8 T cells, while DMPA selectively decreases frequencies of CD4 T cells and enhances percentages of TNF-α+ CD4 T cells. Importantly, we show that DMPA treatment, but not NET-EN, diminishes mucin production and higher mucin, cell-associated mucin in particular, in NET-EN treated mice correlated with lower HSV-2 infection in the vaginal tract.

## 2 Materials and methods

### 2.1 Mice

Eight- to ten-week-old C57BL/6 female mice were purchased from Charles River Laboratories (Saint-Constant, Quebec, Canada). All mice were maintained under specific-pathogen-free and standard temperature-controlled conditions that followed a 12 h light and 12 h dark cycle. To ensure that mice remained specific pathogen free, routine quality assurance was conducted by serology and PCR, and this included testing dirty bedding, sentinels, direct resident animals, and exhaust air duct samples of racks. All animal studies were approved by, and in compliance with, the Animal Research Ethics Board at McMaster University in accordance with the Canadian Council of Animal Care guidelines.

### 2.2 Ovariectomy surgeries

To remove the influence of any endogenous hormones in mice they underwent ovariectomy (OVX), according to previously published protocols (43). Briefly, mice were administered an injectable anesthetic preparation of ketamine and xylazine intraperitoneally. Ovaries were removed by making two bilateral incisions, followed by small incisions through the peritoneal wall, and excising them through the incisions. Peritoneal muscle incisions were closed by sutures and subcutaneous incisions were closed using surgical clips. All mice recovered for 10 to 14 days before the start of experiments.

### 2.3 Hormone treatments and measurement

**Figure 1:**
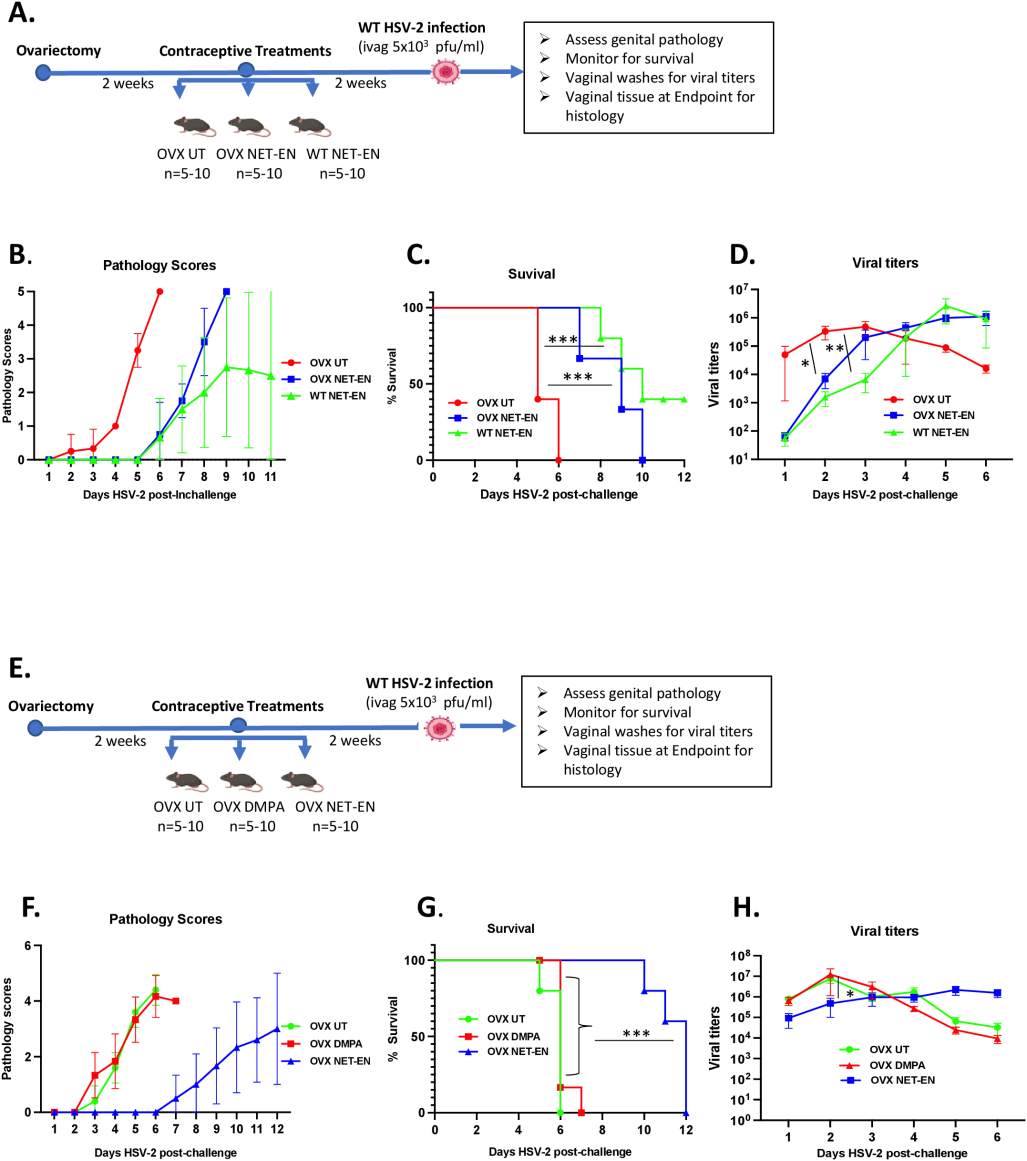
Analysis of survival rates, pathology and HSV-2 virus titers in the vaginal tract following NET-EN, DMPA, or no treatment of WT naïve and OVX mice. C57BL/6 wild-type or OVX mice (n=5/group) were treated with NET-EN, DMPA or were untreated (UT) for 2 weeks prior to intravaginal challenge with HSV-2 (5 x 10^3^ PFU/mouse). After challenge, vaginal washes were collected daily for up to 6 days to determine viral titers. Mice were also monitored for survival, and pathology scores recorded daily for up to 12 days. **(A)** Schematic depiction of experimental design. **(B)** Average pathology scores for OVX UT, OVX NET-EN and WT NET-EN treatment groups are depicted. **(C)** Survival of mice among the aforementioned treatment groups. **(D)** Data shown represent the average viral load per group (mean ± SEM) for each day. **(E)** Schematic demonstration of experimental design. **(F)** Average pathology scores for OVX UT, OVX DMPA and OVX NET-EN treatment groups are shown. **(G)** Survival of mice among OVX UT, OVX DMPA and OVX NET-EN treated groups. **(H)** Data shown represent the average viral load per group (mean ± SEM) for each day. Significance of difference in survival **(C, G)** was calculated using the log rank (Mantel-Cox) test (***, P < 0.001). Significance of differences in viral load **(D, H)** were analyzed using a one-way ANOVA with Tukey’s multiple-comparison test. *, P < 0.05, **, P<0.01.

To examine the effect of NET-EN and DMPA, two weeks after OVX, mice were treated by subcutaneous injection of Medroxyprogesterone acetate (DMPA, 2 mg) and subcutaneous insertion of NET-EN hormone pellets (2.5 mg) (experimental design in **Figure 1E**) manufactured by Innovative Research of America (Sarasota, FL, USA), according to previously published protocols (43). In a separate sets of experiments (experimental design in **Figure 1A**), a group of wild-type naïve C57BL/6 mice and OVX mice were implanted with NET-EN 2.5 mg pellets subcutaneously as a hormone intact model to represent the preclinical scenario in fertile women who receive hormonal contraceptive. These hormone pellets are designed to release 476 ng/day/mouse for 21 days. Briefly, 2 weeks following OVX surgery, mice were anesthetized with isoflurane and implanted subcutaneously with NET-EN pellets. The level of serum MPA and NET resulting from this dose are shown in **Supplementary Figure S1**. These serum levels correspond to those shown previously with physiological doses of DMPA and NET-EN in women (44, 45).

NET and MPA concentrations in serum samples were measured by a combination of Ultra Performance Liquid Chromatography paired with Mass Spectrometry (UPLC-MS) at the Small Molecule Biomarker Core facility at the University of Pittsburgh School of Pharmacy (Pittsburgh, Pennsylvania, USA), as described previously (46). Briefly, Testerone-d3 was added as in internal control to each serum samples and liquid-liquid extraction performed with N-butyl chloride. After centrifugation (4000 RPM) the supernatants were collected and dried with nitrogen at 40 deg C and then reconstitution in 50:50 acetonitrile:water. An Acquity UPLC system with BEH C18 column (Waters) to perform a gradient liquid chromatography. A TSQ Quantum Ultra triple quadrupole mass spectrometer (Thermo Scientific) with a heated electrospray ionization (HESI) source was used to perform Tandem mass spectrometric (MS/MS) analyses. Data acquisition and processing was performed with Xcalibur software v4.0 (Thermo Scientific, San Jose, CA, USA).

### 2.4 Intravaginal HSV-2 challenge

Hormone treated mice were challenged intravaginally with HSV-2. Two weeks after hormone treatments, mice were anaesthetized intraperitoneally with ketamine/xylazine and infected with 10 µL WT HSV-2 strain 333 (5x10^3^ PFU/mouse) intravaginally. After inoculation, mice were placed on their backs for approximately 30 to 45 min to allow for the inoculum to infect the vaginal tract.

### 2.5 Collection of vaginal washes

Vaginal washes were collected for up to 6 consecutive days post-challenge by pipetting 30 µL of phosphate-buffered saline (PBS) into and out of the vagina 5 to 6 times. This was repeated twice to collect a total volume of 60 µL/mouse that was stored at -80°C until required.

### 2.6 Genital pathology scoring

Genital pathology was monitored daily post-challenge based on a five-point scale, as described previously (47): no infection (0), slight redness of external vagina (1), swelling and redness of vagina (2), severe swelling and redness of vagina and surrounding tissues (3), genital ulceration with severe redness and hair loss (4), and severe ulceration extending to surrounding tissues, ruffled hair, and lethargy (5). Animals were sacrificed before they reached stage 5. To compare groups, cumulative pathology scores were determined by tabulating the number of mice with the highest score of pathology they achieved and the number of days that score was observed. Mice that did not survive the challenge/reached endpoint and had to be euthanized were given the highest pathology score for the duration of the experiment to accurately reflect overall pathology for each group. The sum of these scores for all the mice was the total level of pathology for each group, and the average pathology score per mouse for each group was then calculated.

### 2.7 Viral titration

Viral shedding in vaginal washes was determined by conducting viral plaque assays on Vero cell (ATCC, Manassas, VA) monolayers, as described previously (47). Vero cells were grown in α-MEM (Gibco Laboratories, Burlington, ON, Canada) supplemented with 5% fetal bovine serum (FBS; Gibco Laboratories), 1% penicillin-streptomycin (Invitrogen, Burlington, ON, Canada), L-glutamate (Bio-Shop Canada Inc., Burlington, ON, Canada), and 1% HEPES (Invitrogen). Cells were grown to confluence in 12-well plates, and vaginal washes were serially diluted in serum-free α-MEM and then added to monolayers. Infected monolayers were incubated at 37°C for 2 h with intermittent shaking in every 20 to 30 minutes and then overlaid with 10% FBS-α-MEM. The plates were then incubated at 37°C for 48 h to allow infection to occur. Next, cells were fixed and stained with crystal violet, and viral plaques were enumerated under a microscope. The number of PFU per milliliter was calculated by taking a plaque count for every sample and accounting for the dilution factor.

### 2.8 Single cell isolation from tissues and cultures

Spleen cells were isolated by mechanically disrupting the spleen on a 40 µm filter a 6-well plate, centrifuged and red blood cells lysed by ACK lysis buffer (0.15 M Ammonium chloride, 0.01M Potassium bicarbonate, 0.0001 M Disodium EDTA in 1 L distilled water (pH 7.2), our laboratory made). The remaining cells were washed and resuspended in RPMI 1640 medium supplemented with 10% FBS, 100 IU/ml penicillin, 100 g/ml streptomycin, 1% L-glutamine, 0.1% 2-mercaptoethanol, 1% nonessential amino acids, and 1% sodium pyruvate (Gibco Life Technologies, Burlington, ON, Canada). For vaginal tissue analysis, vaginal tracts were removed, pooled, cut lengthwise, washed to remove mucous, and minced into 2- to 4 mm pieces. The vaginal tissue pieces were enzymatically digested in 15 ml of RPMI 1640 containing 0.00157 g/ml collagenase A (Roche Life Science, USA) at 37°C on a stir plate for 2 h and filtered through a 40 µm filter to obtain a total tissue cell suspension (48). Vaginal cell samples were then centrifuged for 10 min (1,200 rpm) at 4°C.

Single-cell suspensions from different tissues were resuspended in 1 to 5 ml of RPMI medium. Finally, mononuclear cells were counted, and cell preparations were seeded in a 96-well plate at 1 x 10^6^ cells/well for spleen and 5x10^5^ cells/well for vaginal samples; the total volume in the well was topped up to 200 µL with complete RPMI 1640 medium. Cells were either left unstimulated or cultured with a T cell stimulation cocktail plus protein transport inhibitors (500x) (cocktail of PMA, ionomycin, brefeldin A, and monensin (eBioscience, San Diego, CA, USA) for up to 16 h.

### 2.9 Flow cytometry

Following 12 to 16 h of incubation with the stimulation cocktail at 37°C, cells were collected, washed and incubated for 15 min with 50:l of Fc block (anti-mouse CD16/32; BD Bioscience) to reduce nonspecific Fc receptor staining. Cells were then stained for cell surface markers using the following antibodies at concentrations based on manufacturer’s instructions: anti-CD4-PerCP-Cy5.5, anti-CD3-PE-CF594 and anti-CD8-BV786 (Invitrogen). Cells were incubated with these antibodies for 30 min and washed. Cells were then stained with allophycocyanin (APC)-ef780 viability dye (eBioscience) for 20 mins and washed. Cells were permeabilized and fixed for 20 mins using the BD Cytofix/Cytoperm buffer set (BD Biosciences) according to the manufacturer’s protocol. Cells were then stained for intracellular markers using the following antibodies: anti-IFN-γ-BV421 and anti-TNF-α-FITC (Invitrogen). All antibodies were validated and titrated for optimal conditions before their application in the experiments. The accuracy of staining positive cutoffs was verified by fluorescence minus one (FMO) controls. Data was collected for mononuclear lymphocytes populations isolated from spleen and vaginal tract by flow cytometric analysis using CytoFLEX flow cytometer system (Beckman Coulter Life Sciences, Indianapolis, USA), and results were analyzed using FlowJo software (Tree Star, Ashland, USA).

### 2.10 Histology, PAS staining and Muc1 protein and HSV-2 Immunohistochemistry

Vaginal tissues were collected from OVX mice after 1 week and 3 weeks of NET-EN and DMPA treatments, and at the end point following HSV-2 challenge. Tissues were fixed in 10% formalin or methacarn solution (60% methanol, 30% chloroform and 10% glacial acetic acid) for 48-72 h. Vaginal tracts were then cut into two pieces, placed in cassettes and transferred to 70% ethanol. The McMaster Immunology Research Center (MIRC) Histopathology Core Facility processed tissues and performed, PAS (Periodic Acid Schiff) staining, Muc1 and HSV-2 immunohistochemical staining. Briefly, Muc1 protein was detected in vaginal tissue sections with Muc1 monoclonal hamster antibody (MH1, CT2, Invitrogen, ThermoFisher Scientific, Canada) and HSV-2 was detected by monoclonal mouse anti-HSV-2 antibody (AP1 (B019) (Novus Biologicals, Cedarlane, Burlington, Canada) staining following antigen retrieval in citric acid using a standard biotin-streptavidin detection system and 3,3’-diaminobenzidine tetrahydrochloride as chromagen, then counter staining with hematoxycilin. Slides were scanned using the Leica Aperio Scanscope XT and viewed using Aperio ImageScope software. In order to verify the specificity of the Muc1 antibody in our system, we ran positive and negative controls (49) by testing vaginal tissue sections collected from normal mice in estrus as negative control and that of normal mice in diestrus as positive control. Signal intensities of both PAS and Muc1 images were quantified using Fiji ImageJ software (NIH, Bethesda, MD, USA).

### 2.11 Muc1 ELISA

Vaginal washes were collected from OVX mice one week or three weeks after placebo, NET-EN or DMPA treatments and stored at -80 °C. Muc1 concentrations in the vaginal washes was measured by mouse Muc1 ELISA kit (Novus Biologicals, Cedarlane, Burlington, ON) according to the manufacturer’s instruction.

### 2.12 Statistical analysis

Statistical analysis and graphical representation were performed using Graph-Pad Prism 10 (GraphPad Software, San Diego, CA). The Mantel-Cox log rank test was used to calculate significant differences in survival. Data are expressed as means with standard errors of means (SEMs) error bars, typically derived from a minimum of 3 independent experiments, with n=5-10 mice/group. Significance was calculated by comparing means using one-way analyses of variance (ANOVA) or t tests, with appropriate additional tests, as indicated in the figure legends. Data were considered statistically significant if the P values were <0.05. Significant differences are indicated as *P<0.05, **P<0.01, ***P<0.001 or, ****P<0.0001.

## 3 Results

### 3.1 NET-EN treatment extends survival and delays genital pathology in OVX mice intravaginally infected with HSV-2, in comparison to DMPA treatment

We and others have previously reported that DMPA-treated mice show enhanced genital pathology and mortality following wild type (WT) HSV-2 virus challenge (4, 5, 50–52). Studies have shown that DMPA enhances the susceptibility of wild-type mice to genital HSV-2 infection by disrupting mucosal barrier functions (16, 50) and a more recent study found comparable mortality rates and increased genital mucosal permeability between DMPA versus NET-EN treated wild-type mice, but slower onset of genital pathology with NET-EN treatment (4).

In this study we compared the effects of NET-EN and DMPA on intravaginal HSV-2 infection utilizing ovariectomized (OVX) mice to exclude any internal hormonal influence. To optimize delivery of physiologically relevant doses of hormones, we compared serum levels in mice at varying doses (**Supplementary Figures S1A, B**). Of note, NET or MPA was not detected in serum samples of OVX untreated mice that we used as negative controls by the highly sensitive and robust hormonal assay system (**Supplementary Figures S1A, B**). The levels of hormone were comparable to that found in women using those contraceptives, based on published pharmacokinetic studies for NET-EN and DMPA in women that showed serum NET concentration of ~33 ng/ml and MPA concentration level 2.3-4.6 ng/ml (44, 45). Thus, these doses were used throughout this study: NET-EN (2.5 mg pellet, 21-day release) and DMPA (2 mg s.c. injection).

Experimental designs for two separate experiments are depicted in **Figures 1A, E**. OVX mice were subcutaneously treated with NET-EN or DMPA and compared to untreated (UT) controls (**Figure 1E**). In a separate set of experiment, wild-type naïve mice and OVX mice were treated subcutaneously with NET-EN to compare the effects of NET-EN in hormone intact versus hormone deficient mice and a group of OVX untreated mice used as controls (**Figure 1A**). Two weeks later, mice were challenged intravaginally with HSV-2 (5x10^3^ pfu), and then monitored for survival, genital pathology and with vaginal washes collected to assess viral titers. Although the major purpose of this study was to understand the mechanistic insights as to how hormonal contraceptive NET-EN regulates the susceptibility to HSV-2 infection compared to well-studied DMPA, we first assessed the impact of NET-EN on wild-type naïve mice versus OVX mice (experimental design shown in **Figure 1A**). Here we observed lower levels and delayed genital pathology starting at day 5 post-challenge and 40% survival in NET-EN treated wild-type mice. NET-EN treated OVX mice showed delayed onset of genital pathology (day 5) similar to WT naïve mice, however, all mice succumbed to infection by day 10 post-challenge. In contrast, OVX untreated control mice showed genital pathology as early as day 2 and all mice reached end-point by day 6 post-challenge (**Figures 1B, C**). Further, NET-EN treated WT and OVX mice showed significantly lower virus shedding on day 2 but higher shedding on day 5/6 post challenge compared to OVX UT mice (**Figure 1D**).

We then evaluated the effects of NET-EN and DMPA in OVX mouse model (experimental design depicted in **Figure 1E**). We found that NET-EN-treated mice had a delayed onset of genital pathology starting around day 6 post-challenge and survived up to day 12 (20% mortality at day10 and day 11, and 60% mortality at day 12) (**Figures 1F, G**). In contrast, DMPA-treated mice and untreated control mice developed genital lesions starting from day 2 and all mice reached endpoint by day 7 (DMPA group) or day 6 (UT OVX) post-challenge (**Figures 1F, G**). The differences seen in pathology and mortality time course in NET-EN and DMPA-treated mice was clearly quantitated by the calculated cumulative pathology scores shown in **Table 1**. The untreated and DMPA-treated mice had similar scores (31.8 and 30, respectively) while NET-EN was much lower (7.0). Vaginal washes collected daily from days 1 to 6 post-challenge were assessed for viral titers. NET-EN-treated mice exhibited significantly lower virus shedding at day 2 post-challenge compared to DMPA- and untreated mice that showed the highest virus titers on day 2 (**Figure 1H**). Collectively, NET-EN-treated OVX mice show delayed mortality, delayed onset of genital pathology and delayed viral shedding after primary HSV-2 infection compared to DMPA treated mice.

**Table 1:** Cumulative pathology scores^1^ 12 days after HSV-2 challenge of untreated, DMPA and NET-EN treated OVX mice (n=5 mice per treatment groups).

Group	Highest Pathology Score	Number of Mice	Number of Days	Cumulative Pathology Score	Average Pathology Score per mouse
Mock	5 5 4	1 1 3	8 7 7	40 35 84	31.8
DMPA	5 4 4	2 3 1	7 7 6	70 84 24	30.0
Net-EN	5 4 4	1 1 3	3 2 1	15 8 12	7.0

^1^ Number of days in the table indicate total number of days counted from the day when a mouse reached the endpoint up to day 12 post HSV-2 infection (the last date of endpoint monitoring).

### 3.2 NET-EN and DMPA have differential effects on the frequencies and numbers of CD4 and CD8 T cells in the vaginal tract

**Figure 2:**
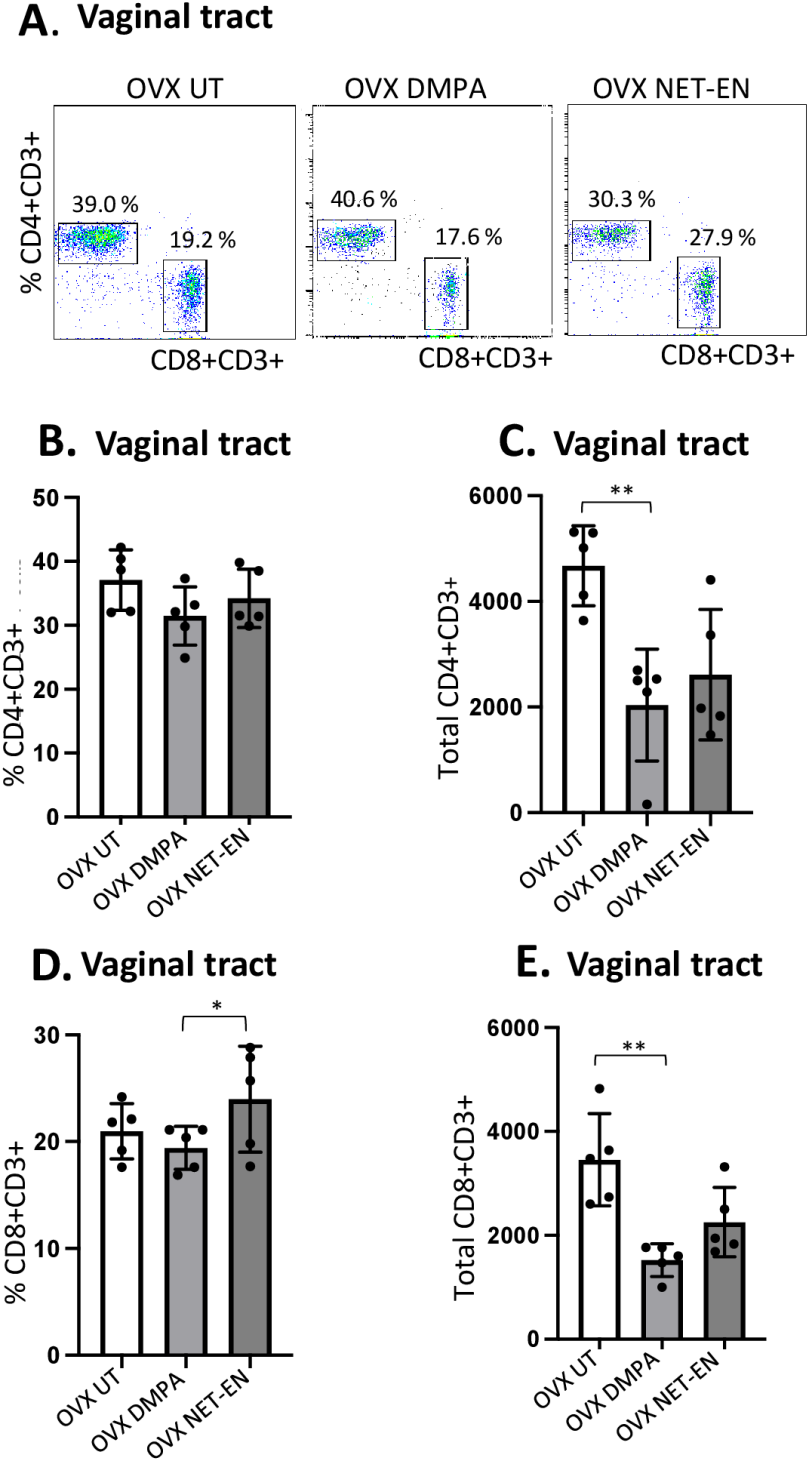
Analysis of vaginal tract CD4 and CD8 T cell frequencies in NET-EN-treated, DMPA-treated and untreated OVX mice. OVX mice were treated with NET-EN (2.5 mg) or DMPA (2 mg) or left untreated (UT) for 3 weeks. Mice were then sacrificed, VT tissues were collected, and single cell suspensions were isolated. The cells were then stimulated with the T cell stimulation cocktail up to 16 h, followed by fluorescence antibody staining for extracellular markers including CD3, CD4 and CD8 prior to analysis by flow cytometry. A primary gating strategy (**Supplementary Figure S3**) allowed analysis of viable, single CD3 positive lymphocytes. **(A)** Representative dot plots showing identified CD4+ and CD8+ T cells. **(B)** Bar graph shows the percentage of viable CD4+ T cells, mean ± SEM (n = 5). **(C)** Bar graphs displaying the total number of CD4+ T cells per vaginal tissue, mean ± SEM (n = 5). **(D)** Bar graphs showing the percentage of CD8+ T cells, mean ± SEM (n = 5) and **(E)** total number of CD8+ T cells, mean ± SEM (n = 5), for each vaginal tissue isolated from NET-EN, DMPA or untreated (UT) mice. Bars indicate mean ± SEM. All data were collected from 3 independent experiments. Data were analyzed utilizing the one-way ANOVA with Tukey’s multiple comparison test. *, P <0.05; **, P <0.01.

Recent murine model studies indicate that both DMPA and NET-EN increase genital mucosal permeability to activated leukocytes, although the effect of NET-EN was less pronounced (4). A few human studies have shown that DMPA enhances T cell IFN-γ and TNF-α production (22, 23), but others found no difference between NET-EN and DMPA on T cell responses in women (21). Definitive data on the effects of DMPA or NET-EN on frequencies and functions of CD4 and CD8 T cells, particularly in murine model is lacking. To address the paucity of data on T cell responses with NET-EN and DMPA, we compared their effects on CD4 and CD8 T cell frequency, number and cytokine responses in mice, both in local FRT tissue and systemic spleen tissue. Ovariectomized mice were treated subcutaneously with DMPA (2 mg) or NET-EN (2.5 mg) and 3 weeks later cells were isolated from the vaginal tract and were stimulated with PMA/Ionomycin and analyzed by flow cytometry for cytokine production (protocol described in **Supplementary Figure S2**). For analysis, cells were first gated on lymphocyte populations, followed by single cells and finally gated on live lymphocytes expressing CD3 (gating strategy depicted in **Supplementary Figure S3**). Next, CD3+ T cell populations were further gated to identify the percentages of CD4+ and CD8+T cells present in the vaginal tract (**Figure 2A**). Total counts of CD8 or CD4 T cells for each subpopulation were calculated based on the number of viable cells per mL of isolated VT cells run on flow cytometer, times the number of viable CD4 or CD8 T cells displayed in the flow cytometry result (31).

Neither DMPA nor NET-EN treatment caused any noticeable changes in frequencies of CD4 T cells in the vaginal tissue (**Figure 2B**), but DMPA resulted in significant depletion of total number of CD4 T cells present in the vagina, compared to untreated control group (**Figure 2C**). Although neither NET-EN or DMPA treatments altered the frequency of CD8 T cells relative to control, NET-EN-treatment resulted in slightly higher frequency of CD8 cells than DMPA-treatment (**Figure 2D**). A reduction in total numbers of CD8 T cells in the vaginal tract was evident for both NET-EN and DMPA treatment groups compared to untreated control group, but this only reached statistical significance for the DMPA treatment group (**Figure 2E**).

### 3.3 NET-EN and DMPA have differential effects on the frequencies and numbers of IFN-γ and TNF-α producing CD4 and CD8 T cells in the vaginal tract

**Figure 3:**
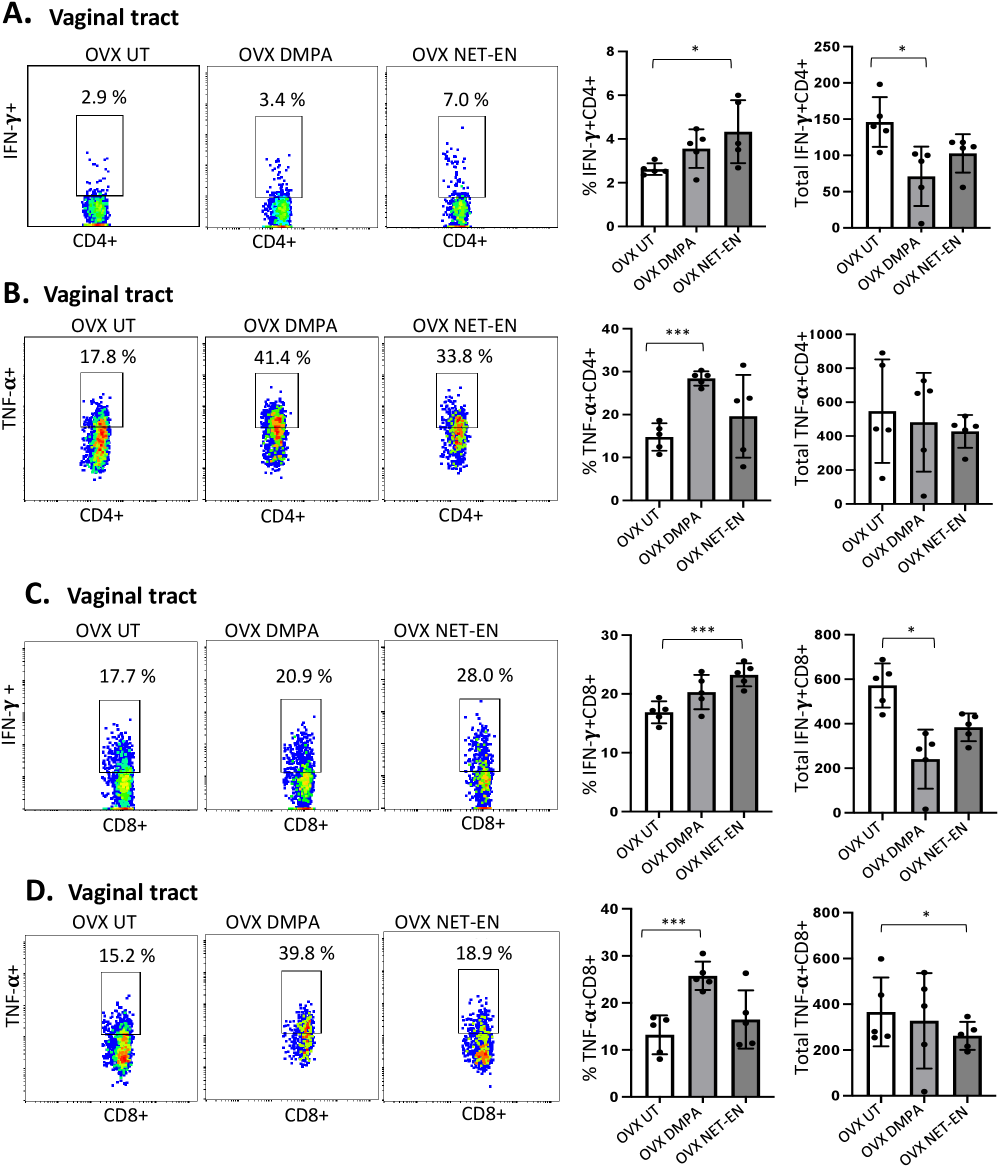
Analysis of vaginal tract CD4 and CD8 T cells for IFN-γ and TNF-α expression in NET-EN-treated, DMPA-treated and untreated OVX mice. OVX mice were treated with NET-EN (2.5 mg) or DMPA (2 mg) or left untreated (UT) for 3 weeks (n=5 per group). Mice were then euthanized, vaginal tracts tissues were collected, and single cell suspensions were isolated. The cells were then stimulated with the T cell stimulation cocktail up to 16 h, followed by fluorescence antibody staining for extracellular markers such as CD3, CD4 and CD8 and intracellular staining for cytokines IFN-γ and TNF-α prior to analysis by flow cytometry. A primary gating strategy (**Supplementary Figure S3**) allowed analysis of viable, single CD3 positive lymphocytes, then gated for CD4+ and CD8+ cells. **(A)** Representative dot plots showing the gated CD4+ T cells and their IFN-γ fluorescence. Inset rectangle identifies IFN-γ+ CD4 T cells with % positive shown. Bar graphs on the right display the mean ± SEM (n = 5) for the percentage of IFN-γ+ CD4 T cells (left panel) and total number of CD4+ IFN-γ+ T cells (right panel). **(B)** Representative dot plots showing the gated CD4+ T cells and their TNF-α fluorescence. Inset rectangle identifies TNF-α+ CD4 T cells with % positive shown. Bar graphs display the mean ± SEM (n = 5) for the percentage of TNF-α+ CD4 T cells (left panel) and total number of TNF-α+ CD4 T cells (right panel). **(C)** Representative dot plots showing the gated CD8+ T cells expressing IFN-γ. Inset rectangle identifies CD8+IFN-γ+ cells with % positive shown. Bar graphs show the mean ± SEM (n = 5) for the percentage IFN-γ+ CD8 T cells (left panel) and total number of IFN-γ+ CD8 T cells (right panel). **(D)** Representative dot plots display the gated CD8+ T cells expressing TNF-α. Inset rectangle identifies TNF-α+ CD8 T cells with % positive shown. Bar graphs show the mean ± SEM (n = 5) for the percentage TNF-α+ CD8 T cells (left panel) and total number of TNF-α+ CD8 T cells (right panel). All data are drawn from 3 independent experiments. Data were analyzed utilizing the one-way ANOVA with Tukey’s multiple comparison test. *, P <0.05; ***, P <0.001.

We next assessed IFN-γ and TNF-α expression by CD4+ and CD8+ T cells with NET-EN or DMPA treatments. We found a significant selective enhancement in the frequencies of IFN-γ+ CD4 T cells with NET-EN (**Figure 3A**) and TNF-α+CD4 T cells with DMPA treatments in comparison to untreated controls (**Figure 3B**). In terms of absolute cell numbers, there was a significant decrease in IFN-γ+CD4 T cells with DMPA treatment compared to untreated controls. NET-EN treatment displayed a trend toward reduce numbers of IFN-γ+CD4 T cells, that did not reach significance (**Figure 3A**). Significant increase in frequency of IFN-γ+CD8 T cells was seen with NET-EN treatment (**Figure 3C**) and the frequency of TNF-α+CD8 T cells in DMPA treated mice (**Figure 3D**). Collectively, DMPA treatment significantly decreases the number of T cells in the vagina while selectively enhancing the frequency of TNF-α producing CD4 and CD8 T cells. In contrast, NET-EN treatment significantly enhances frequency of IFN-γ producing CD4 and CD8 T cells. **Table 2** summarizes the changes in frequency and total number or CD4 and CD8 T cell subsets isolated from vaginal tissue in the different treatment groups.

**Table 2:** A summary^1^ of results for immune cells response to no hormone treatment (UT), NET-EN vs DMPA treatments (n=5 mice for each treatment groups).

Tissue	T Cell Subsets	Change from untreated vs NET-EN	Change from untreated vs DMPA
	% CD4+	~	~
	Total CD4+	~	**↑↑**
	% IFNγ+ CD4+	**↑↑**	~
	Total IFNγ+ CD4+	~	↓↓
	% TNFα+ CD4+	~	**↑↑**
Vaginal Tract	Total TNFα+ CD4+	~	~
	% CD8+	~	~
	Total CD8+	~	↓↓
	% IFNγ+ CD8+	**↑↑**	~
	Total IFNγ+ CD8+	~	**↑↑**
	% TNFα+ CD8+	~	**↑↑**
	Total TNFα+ CD8+	↓↓	~
	% CD4+	~	↓↓
	Total CD4+	~	~
	% IFNγ+ CD4+	↑↑	~
	Total IFNγ+ CD4+	~	~
	% TNFα+ CD4+	↑↑	↑↑
Spleen	Total TNFα+ CD4+	~	~
	% CD8+	↑↑	~
	Total CD8+	~	~
	% IFNγ+ CD8+	↑↑	~
	Total IFNγ+ CD8+	~	~
	% TNFα+ CD8+	~	~
	Total TNFα+ CD8+	~	~

Symbols indicate: ~ no significant change; ↑↑ significant increase; ↓↓ significant decrease relative to untreated controls.

**Figure 4:**
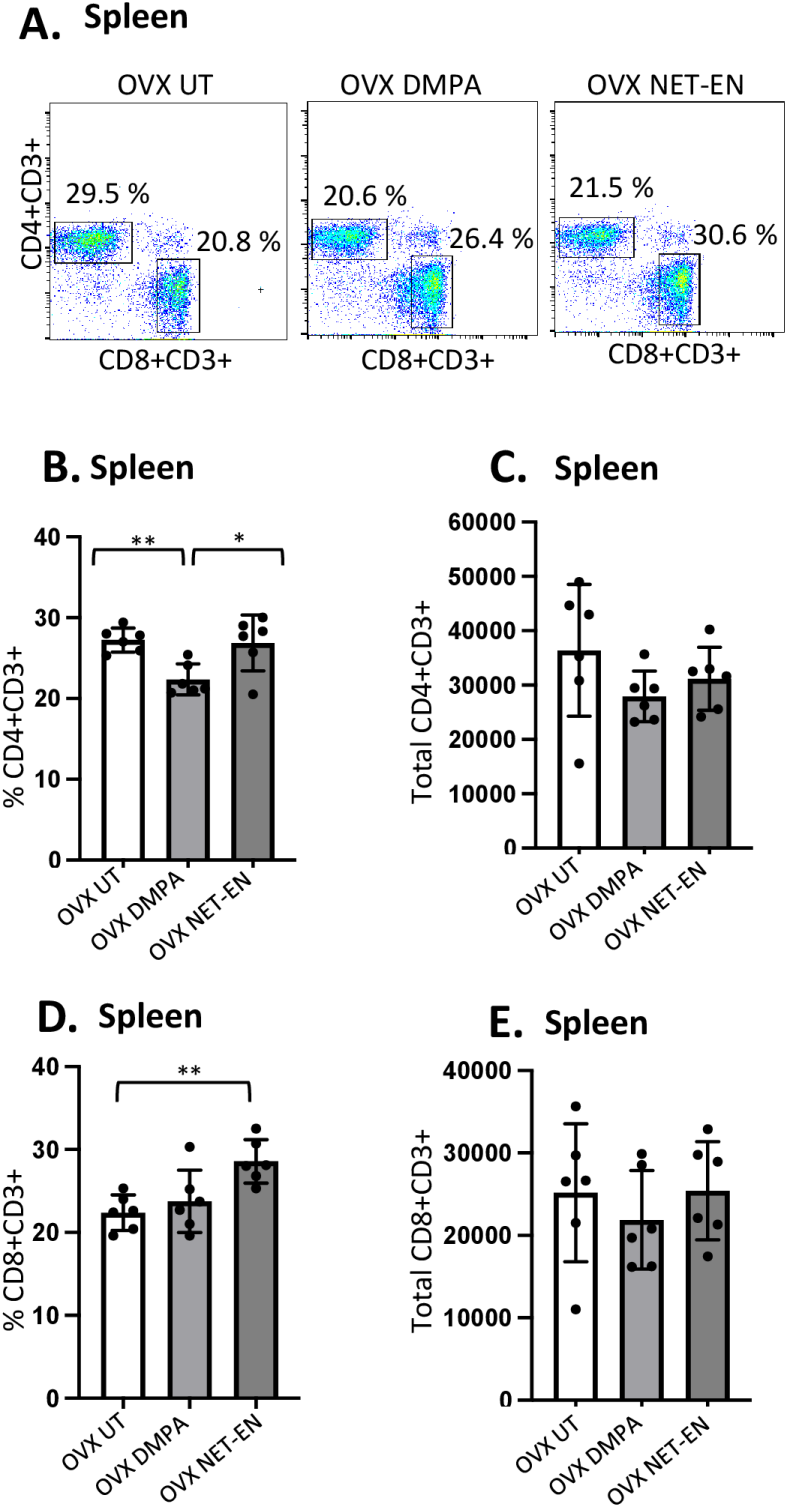
Analysis of splenic CD4 and CD8 T cells populations in NET-EN-treated, DMPA-treated and untreated OVX mice. OVX mice were treated with NET-EN (2.5 mg), DMPA (2 mg) or left untreated (UT) for 3 weeks. Mice were then sacrificed, their spleens collected and single cell suspensions were isolated. Cells were then stimulated with the T cell stimulation cocktail up to 16 h, followed by fluorescence antibody staining for extracellular markers, such as CD3, CD4 and CD8 and analyzed by flow cytometry. A primary gating strategy (**Supplementary Figure S4**) allowed analysis of viable, single CD3 positive lymphocytes. **(A)** Representative dot plots showing identified CD4+ and CD8+ T cells. **(B)** Bar graph shows the mean ± SEM (n = 5) for the percentage of viable CD4+ T cells, and **(C)** the number of viable CD4+T cells in spleen isolated from NET-EN, DMPA or untreated mice. **(D)** Bar graph showing the mean ± SEM (n = 5) for the percentage of CD8+ T cells, and **(E)** the total number of CD8+ T cells in the spleen. All data are drawn from 3 independent experiments. Data were analyzed utilizing the one-way ANOVA with Tukey’s multiple comparison test. *, P <0.05; **, P <0.01.

**Figure 5:**
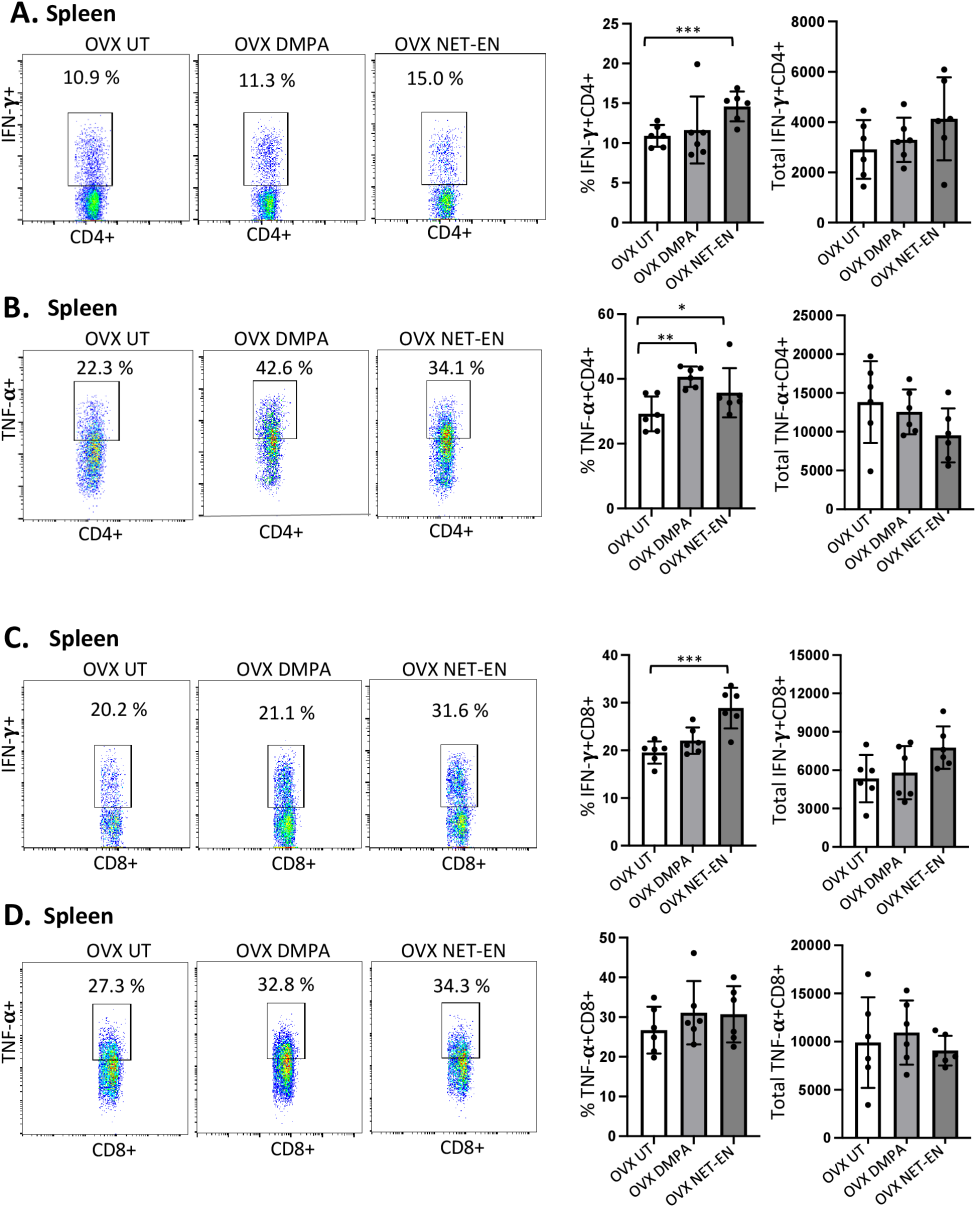
Analysis of splenic CD4 and CD8 T cells for IFN-γ and TNF-α expressions in NET-EN-treated, DMPA-treated and untreated OVX mice. OVX mice were treated with NET-EN (2.5 mg), DMPA (2 mg) or left untreated (UT) for 3 weeks. Mice were then euthanized, their spleens collected and single cell suspensions were isolated. Cells were then stimulated with the T cell stimulation cocktail up to 16 h, followed by fluorescence antibody staining of extracellular markers (CD3, CD4 and CD8) and then intracellular cytokine staining for IFN-γ and TNF-α before analyzed by flow cytometry. A primary gating strategy (**Supplementary Figure S4**) allowed analysis of viable, single CD3 positive lymphocytes and further gated for CD4+ and CD8+ T cell subpopulations. **(A)** Representative dot plots showing the gated CD4+ T cells and their IFN-γ fluorescence. Inset rectangle identifies IFN-γ+ CD4 T cells with % positive shown. Bar graphs on the right display the mean ± SEM (n = 5) for the percentage of IFN-γ+ CD4 T cells (left panel) and total number of CD4+ IFN-γ+ T cells (right panel). **(B)** Representative dot plots showing the gated CD4+ T cells and their TNF-α fluorescence. Inset rectangle identifies TNF-α+ CD4 T cells with % positive shown. Bar graphs display the mean ± SEM (n = 5) for the percentage of TNF-α+ CD4 T cells (left panel) and total number of TNF-α+ CD4 T cells (right panel). **(C)** Representative dot plots showing the gated CD8+ T cells expressing IFN-γ. Inset rectangle identifies CD8+IFN-γ+ cells with % positive shown. Bar graphs show the mean ± SEM (n = 5) for the percentage IFN-γ+ CD8 T cells (left panel) and total number of IFN-γ+ CD8 T cells (right panel). **(D)** Representative dot plots display the gated CD8+ T cells expressing TNF-α. Inset rectangle identifies TNF-α+ CD8 T cells with % positive shown. Bar graphs show the mean ± SEM (n = 5) for the percentage TNF-α+ CD8 T cells (left panel) and total number of TNF-α+ CD8 T cells (right panel). All data are drawn from 3 independent experiments. Data were analyzed utilizing the one-way ANOVA with Tukey’s multiple comparison test. *, P <0.05; **, P <0.01; ***, P <0.001.

^1^ All data are drawn from 3 independent experiments for CD4 and CD8 T cells analyses (**Figures 2**–**5**) and significance were determined analyzing results by the one-way ANOVA with Tukey’s multiple comparison test and P <0.05 was considered significant.

### 3.4 NET-EN and DMPA treatments show distinct effects on the percentages and the numbers of CD4 and CD8 T cells in the spleen

After demonstrating the distinct differences in CD4 and CD8 T cells populations and their functions with NET-EN and DMPA treatments in the local vaginal tract tissue, we compared that response to systemic lymphocyte populations using isolated spleen cells (experimental design depicted in **Supplementary Figure S2**). Spleen cells were first gated on lymphocytes populations, followed by single cells and finally gated on live lymphocytes expressing CD3 (gating strategy shown in **Supplementary Figure S4**). Next, CD3+ T cells were further gated to identify the percentages and absolute numbers of CD4+ and CD8+T cells present in the spleen (**Figure 4A**). It is noteworthy to mention that we found a comparatively higher percentage of double positive CD4+ and CD8+ T cells in the spleen than the vaginal tract which is likely because the spleen has the highest numbers of multiple subsets of immune cells and respond to hormonal treatments with a more diverse magnitude than the vaginal tract cells.

The effects of DMPA and NET-EN on decreasing the overall numbers of T cells seen in the vaginal tract were not seen systemically with splenic lymphocytes. However, the frequency of splenic CD4 T cells was significantly lower with DMPA treatment in comparison to untreated and NET-EN treatments (**Figure 4B**). In contrast, CD8 T cell frequency was higher with NET-EN (**Figure 4D**). Unlike vaginal tissue, the numbers of CD4 (**Figure 4C**) and CD8 T cells (**Figure 4E**) were not different in splenic lymphocytes for DMPA or NET-EN treated mice.

### 3.5 NET-EN and DMPA treatments show distinct effects on the percentages and the numbers of IFN-γ and TNF-α expressing CD4 and CD8 T cells in the spleen

Most mouse model data available so far that demonstrates the effects of sex hormones and hormonal contraceptives mainly focuses on local immune responses in the draining lymph nodes or vaginal tissue. Precise information about the influence of these hormones on systemic immune modulation involving splenic immune cells remain to be studied. We therefore investigated the effects of NET-EN and DMPA on the functional phenotypes of splenic T cells, and compared that with local immune responses the effects on those phenotypes in the VT (experimental design depicted in **Supplementary Figure S2**). We found a greater frequency of IFN-γ producing CD4 cells with NET-EN treatment compared to untreated and DMPA-treated mice (**Figure 5A**). In addition, treatment with either contraceptive resulted in increased frequency of TNF-α+CD4+ T cells with more significant effects using DMPA in comparison to untreated controls (**Figure 5B**). We observed no statistically significant difference in total numbers of splenic IFN-γ or TNF-α expressing CD4+ T cells with either contraceptive. However, the number of IFN-γ+ CD4 T cells showed a trend of increase with NET-EN (**Figure 5A**) and a decrease of TNF-α+CD4 T cells with NET-EN (**Figure 5B**). Importantly, significantly higher frequency of IFN-γ+ CD8 T cells was observed with NET-EN treatment in comparison to untreated controls (**Figure 5C**). However, no difference in frequencies of TNF-α+CD8 T cells were observed for all experimental groups (**Figure 5D**). **Table 2** summarizes the differences in frequency and total number or CD4 and CD8 T cell subsets isolated from spleens in the different treatment groups.

### 3.6 DMPA, but not NET-EN treatment, diminishes mucin production in the vaginal tract

**Figure 6:**
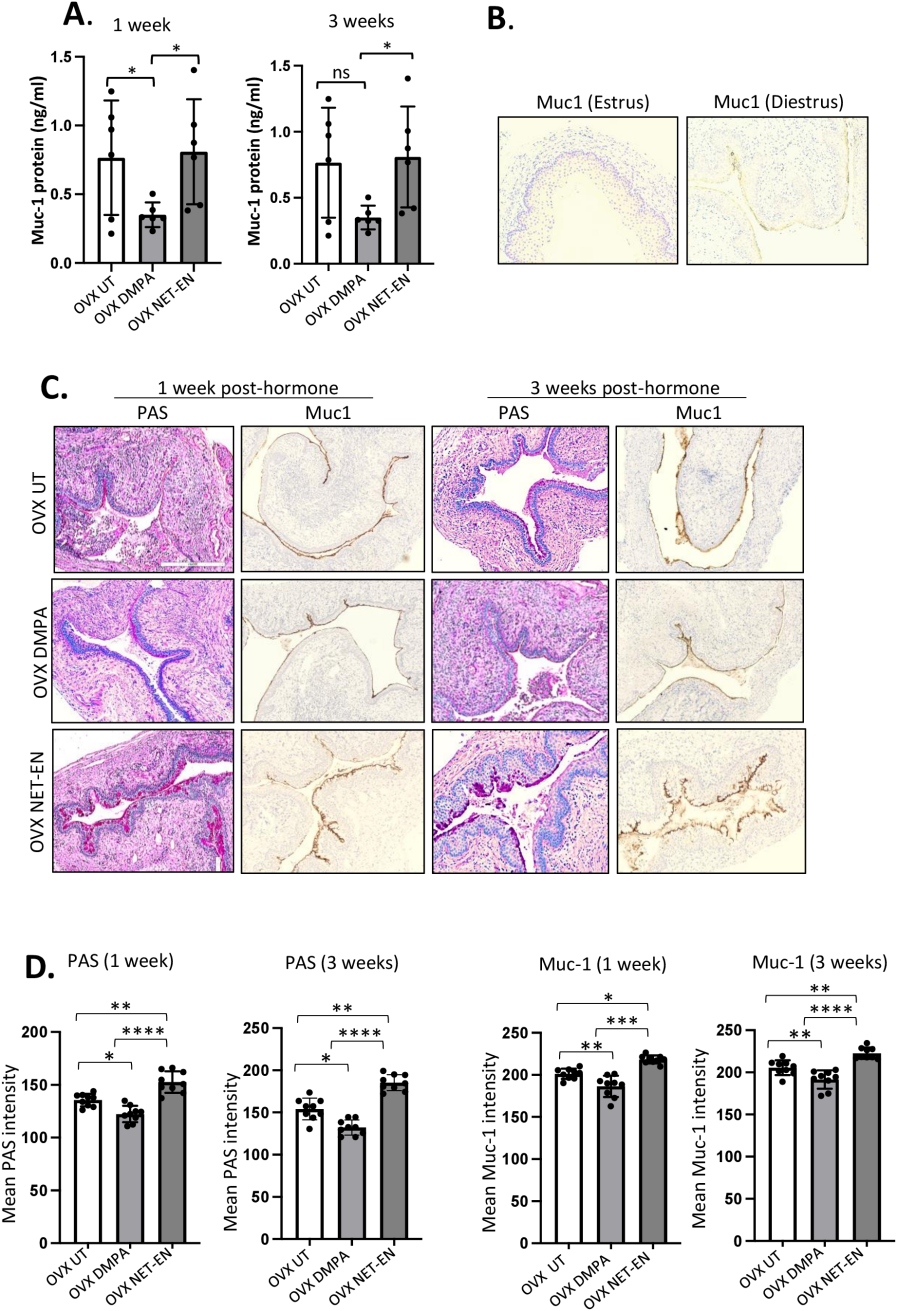
Analysis of mucin production in vaginal tract of OVX mice treated with NET-EN, DMPA or left untreated. OVX mice were treated with NET-EN or DMPA or left untreated (UT) and vaginal washes collected after 1 and 3 weeks of treatment. Some of the treated mice were euthanized and vaginal tissues collected and processed for histological staining with PAS and immunohistochemical staining for Muc1 to detect cell associated Mucin. **(A)** Vaginal washes taken at 1 and 3 weeks were assessed for secreted Muc1 protein levels by commercial mouse Muc1 ELISA kit. Bars indicate mean ± SEM for n = 6 samples. Statistical significance: *, P <0.05. **(B)** Vaginal tissues showing no Muc1 immunohistochemistry staining in vaginal sections taken from estrus stage serves as negative control and Muc1 staining was visible (dark brown) in diestrus used as a positive control. **(C)** Vaginal tissues showing different levels of cell associated mucins (multiple mucin types) as detected by intensity of PAS staining (dark pink) and Muc1 immunohistochemistry staining (dark brown) after 1 and 3 weeks of treatment. **(D)** PAS and Muc1 staining intensities for images of vaginal tissues for different treatments showed in panel **(C)** were quantified using Fiji ImageJ software as depicted in bar graphs. Bars indicate mean ± SEM for n=9 samples (3 images per mouse vaginal tissue, n=3 mice per treatment). All data are drawn from 2 independent experiments. Data were analyzed utilizing the one-way ANOVA with Tukey’s multiple comparison test. *, P <0.05; **, P <0.01; ***, P <0.001; ****, P<0.0001.

In the FRT, the epithelium of the cervix and its glands secrete mucins, which function as a defense against invading pathogen in the uterus and vagina. In this study, we first examined whether NET-EN and DMPA would differentially modulate mucin production in the vaginal tract. OVX mice were treated with NET-EN or DMPA and vaginal washes were collected at 1- and 3-weeks post-treatment (experimental design showed in **Supplementary Figure S2**). Mucin concentration was measured in vaginal washes by ELISA. We detected a significantly diminished level of mucin in the vaginal tract in DMPA treated mice compared with NET-EN treated and untreated OVX mice (**Figure 6A**). In another group of NET-EN and DMPA treated mice, vaginal tissues were harvested at 1 and 3 weeks of treatments, tissues were fixed and subjected to either PAS staining and immunohistochemistry for cell-associated Muc1 protein. The specificity of Muc1 monoclonal antibody staining was verified by staining vaginal tract tissues collected from normal mice in estrus and diestrus as controls (**Figure 6B**). No Muc1 staining was seen in vaginal sections taken from estrus stage and MUC-1 staining was visible in diestrus. Interestingly, NET-EN treated vaginal tissue displayed robust expression of carbohydrates as detected by PAS staining and cell-associated Muc1 protein as detected by immunohistochemistry staining (**Figure 6C**). By contrast, DMPA treatment showed minimal levels of cell-associated Muc1 protein and PAS-stained carbohydrates compared to NET-EN treatment (**Figure 6C**). Of note, OVX mice overall show thinning of vaginal epithelial layer for all treatment groups, however, NET-EN treatment shows increased epithelial layer thickness compared to other groups. Quantification and analyses of both PAS and Muc1 staining images showed significantly higher PAS and Muc1 signal intensities with NET-EN treatment as compared to DMPA and untreated mice. DMPA treatment, on the other hand, showed significant reduction of both PAS and Muc1 signal intensities compared to NET-EN and untreated mice (**Figure 6D**). Thus, these results show strong evidence that DMPA profoundly reduces mucin, whereas NET-EN treatment maintains or enhances mucin in the vaginal tract.

### 3.7 Reduced mucin levels in the vaginal tract with DMPA treatment is associated with increased levels of HSV-2 in contrast to NET-EN treated and untreated mice following HSV-2 infection

**Figure 7:**
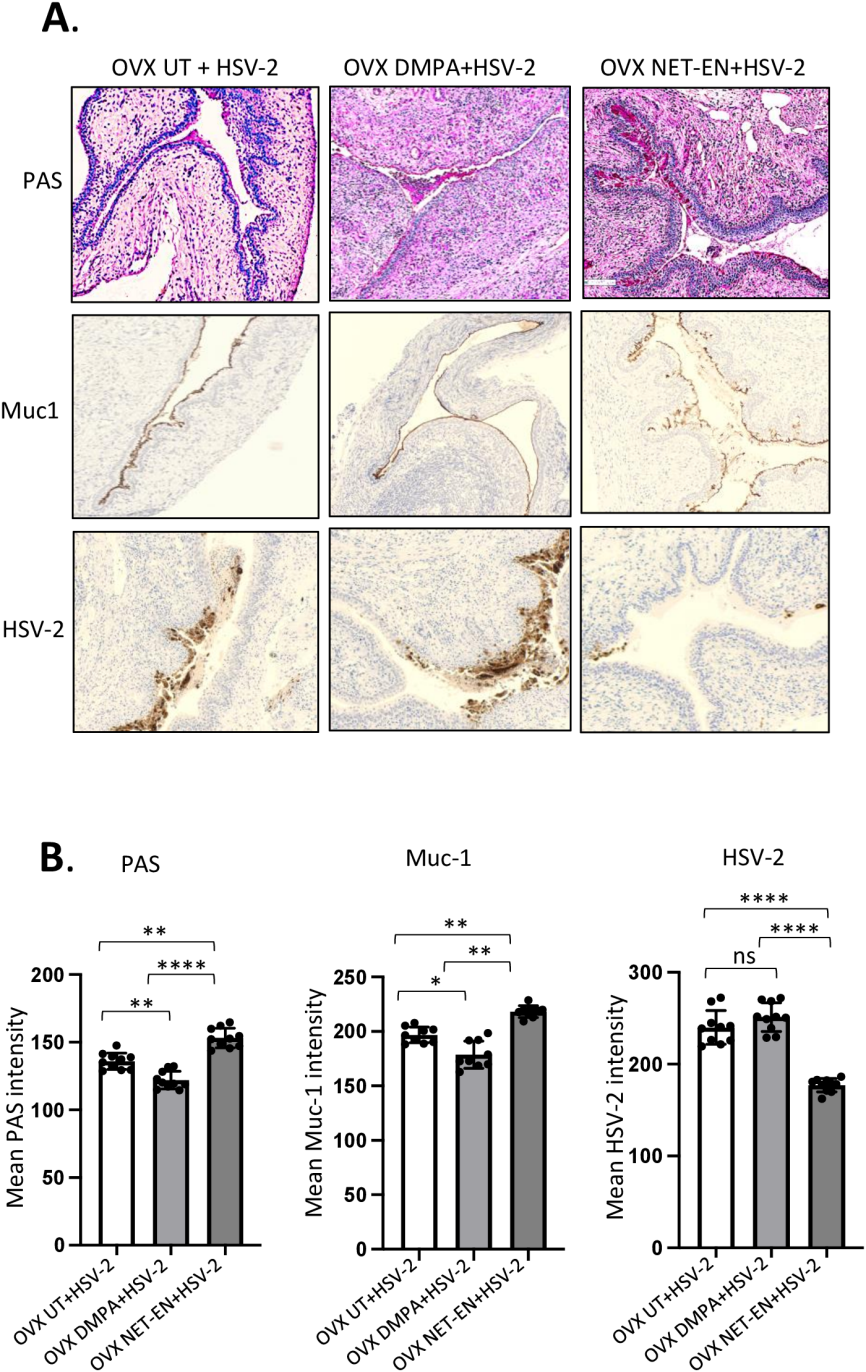
Immuno-histochemical analyses of mucin production and HSV-2 virus detection in the vaginal tract of OVX mice treated with NET-EN, DMPA or left untreated and subsequently infected with WT HSV-2. WT C57BL/6 OVX mice (n=5/group) were treated with NET-EN, DMPA or were left untreated (UT) for 2 weeks and then challenged intravaginally with WT HSV-2 333 (5 x 10^3^ PFU/mouse). Mice were monitored daily for pathology using the 0 to 5 scale (see Methods). Mice for all 3 groups were euthanized at day 5 post HSV-2 challenge when majority of the mice from DMPA and untreated groups reached stage 4/5 HSV-2 pathological score. Vaginal tissues were collected and processed for immuno-histological staining with Muc1 and HSV-2 antibodies. The experiments were repeated twice with similar results and representative slides for different treatment groups are displayed. **(A)** Vaginal tissues showing different levels of cell associated mucins (multiple mucin types) as detected by intensity of PAS staining (top images, dark pink stain) and Muc1 immunohistochemistry staining (middle images, dark brown stain), and HSV-2 immunohistochemistry staining (bottom images, dark brown stain). **(B)** PAS, Muc1 and HSV-2 immunohistochemistry staining intensities for images of vaginal tissues for different treatments exhibited in panel A were quantified by Fiji ImageJ software as shown in bar graphs. Bars indicate mean ± SEM for n=10 images (n=5 mice per treatment and 2 images per vaginal tissue). All data are drawn from 2 independent experiments. Data were analyzed utilizing the one-way ANOVA with Tukey’s multiple comparison test. *, P <0.05; **, P <0.01; ****, P <0.0001.

Next, we investigated whether the relationship between Muc1 production and HSV-2 levels under the influence of NET-EN and DMPA in the vaginal tract. OVX mice were treated with NET-EN or DMPA and then challenged intravaginally with WT HSV-2 (experimental design shown in **Figure 1A**). All mice were euthanized at day 5 post-challenge, before any mouse from any treated group reached stage 5 in pathology, and vaginal tissue were collected and processed for PAS staining and imunohistochemistry staining for HSV-2 or Muc1 protein (**Figure 7A**). We observed pronounced expressions of carbohydrates as detected by PAS staining, as well as significantly increased presence of Muc1 protein in the vaginal tissue only with NET-EN treated mice challenged intravaginally with wild type HSV-2 (**Figure 7A**). Importantly, DMPA treated mice when infected with HSV-2 showed significantly diminished mucin levels in the vaginal tract as detected with PAS and Muc1 protein staining. Quantification of PAS and Muc1 staining images displayed significantly increased levels of both PAS and Muc1 signal intensities in the vaginal tissues of NET-EN treated mice after HSV-2 infection compared to DMPA and untreated mice (**Figure 7B**). In contrast, DMPA treated mice following HSV-2 infection exhibited significantly reduced levels of both PAS and Muc1 signals when compared with NET-EN and untreated mice. Associated with enhanced amounts of cell-associated Muc1 protein, a diminished level of HSV-2 virus particles were detected for NET-EN treatment. Conversely, DMPA and untreated vaginal tissue displayed markedly higher levels of HSV-2 viral particles. Analysis of HSV-2 immunohistochemistry images showed significantly diminished levels of signal intensities in the vaginal tissues of NET-EN treated mice compared to DMPA and untreated mice infected intravaginally with HSV-2, while comparable levels of significantly enhanced HSV-2 signals were observed in DMPA and untreated vaginal tissues (**Figure 7B**, right panel). Of note, we found no significant differences in signal intensities for PAS, or Muc1 between any of the treatment groups when we compared mice that were challenged intravaginally with wild type HSV-2 versus those were not infected within the same hormone treatment (**Supplementary Figure S5**). This indicates a potential role for mucins in inhibiting or delaying infection of the vaginal tract by HSV-2. Collectively, this study provides novel evidence that DMPA treatment decreases Muc1 protein production, while NET-EN may enhance cell-associated Muc1 protein. It follows that the increase in Muc1 expression by NET-EN may result in decreased HSV-2 infection in the vaginal tract of OVX mice.

## 4 Discussion

Depot Medroxyprogesterone (DMPA) and Norethisterone Enanthate (NET-EN) are injectable progestins that have been used as female contraceptives for decades. Although both contraceptives provide effective protection against pregnancy, concerns exist that women using progestin-based contraceptives are more prone to acquiring HIV-1 and other STIs including HSV-2. Despite several meta-analyses and human clinical studies performed in the past decades, this public health issue remains long-standing due to inconsistent and variable findings. In parallel, a few murine model studies have attempted to understand if DMPA and/or NET-EN are associated with increased susceptibility to HIV-1 and HSV-2 infections in wild type intact and humanized mice and the underlying mechanism (4, 5, 53). More comprehensive research is needed to understand and distinguish the effects of DMPA versus NET-EN to determine if progestin-based contraceptives can be used safely without any increase in STI susceptibility.

The main goal of this study was to examine the distinct effects of NET-EN and DMPA on vaginal mucosa and connect the underlying mechanisms along with the susceptibility to genital HSV-2. In order to elucidate the effect of each hormonal contraceptive individually, we used hormone deficient OVX mouse model in order to exclude any endogenous hormonal influence. However, to validate the effects of NET-EN in wild-type hormone intact mice (relevance to fertile women) in our hand, we first examined the susceptibility of wild-type hormone intact mice versus OVX mice treated with physiologically relevant doses of NET-EN (2.5 mg) for two weeks followed by intravaginal WT HSV-2 challenge. OVX UT control mice displayed early onset of genital pathology and they reached end-point by Day 6 post-challenge, by contrast, OVX NET-EN and WT NET-EN mice had comparable genital pathology (day 5 post-challenge) and delayed viral shedding (day 5/6 post-challenge) with WT NET-EN mice showed longer survival (40% survival) than OVX NET-EN mice as they reached endpoint by day 10 post-challenge. These results differ to some extent from the recent study of Quispe Calla et al. (4) which could be due methodological variations (5X daily injections of hormones versus implants), and HSV-2 and NET-EN dose differences. However, our results were similar to the previous study in that NET-EN mice had better outcomes in pathology and survival. We next investigated the susceptibility to HSV-2 infection in the OVX mouse model by treating mice with physiologically relevant doses of NET-EN (2.5 mg) and DMPA (2 mg) (**Supplementary Figure S1**) for 2 weeks and subsequently challenged with WT HSV-2. In comparison to NET-EN treated mice, DMPA treated mice show early onset of severe genital pathology, significantly higher viral titers and succumbed to infection by day 5/6 post-challenge. NET-EN treated mice, on the other hand, show delayed onset of genital pathology, less virus shedding and longer survival up to 12 days. Our findings differ from an intact mouse model study demonstrating that DMPA and NET-EN treated mice displayed comparable mortality and morbidity after HSV-2 challenge, but greater pathology in DMPA than NET- EN treated mice (4). Possible reasons for differences of findings between our study and the aforementioned study could be attributed to differences in experimental methods. Importantly, we used OVX mice in our studies compared to normal WT C57BL/6J mice used in the other study. In addition, there were differences in dose, frequency and route of contraceptives administration, duration of treatment and doses of HSV-2 virus challenge. Of note, we have optimized NET-EN and DMPA doses by administering different doses in OVX mice and then measured serum levels of NET and MPA (**Supplementary Figure S1**), to ensure the doses were comparable to physiological concentrations found in women (4).

In other work, Ray et al. (11) demonstrated that physiologically relevant doses of DMPA, but not NET-EN, significantly increased R5-tropic HIV-1 replication ex vivo in human endocervical and ectocervical explant tissue culture. This involved a mechanism where MPA acted through the glucocorticoid receptor (GR) to increase the frequency of HIV-1 target cells. Our group previously demonstrated that DMPA increased intravaginal HIV-1 acquisition 3.25-fold in humanized mice through mechanisms including enhancing HIV-1 target cells, disrupting vaginal epithelial barrier functions and inducing hypo-estrogenic condition in mice similar to that seen in women (5). Unlike MPA, NET has lower affinity for the progesterone receptor (PR) (3), and a portion of NET is aromatized *in vivo* to ethinyl estradiol, a potent estrogen with high estrogen receptor affinity (54). Estrogen is known to have a protective effect against sexually transmitted viruses, including HSV-2 and HIV-1 (30, 31, 55). Quispe Calla et al. demonstrated that susceptibility of DMPA-treated humanized mice to genital infection with cell-associated HIV-1 was eliminated by subsequent treatment with exogenous estrogen (53). It was established that aromatic conversion of NET *in vivo*, turns NET-EN more estrogen-like and less progestin-like, but similar alteration of DMPA does not occur (54). This may imply a possible mechanism as to why we observed decreased susceptibility to HSV-2 infection with NET-EN, but not with DMPA, as we have previously reported similar but stronger protection with E2 treatment (30, 55). Our study here has re-affirmed that DMPA causes increased susceptibility to HSV-2 infection and indicates that NET-EN should be examined further as a safer contraceptive choice compared to DMPA, with respect to sexually transmitted and blood borne infections (STBBI) risk.

Hormonal contraceptives are known to modulate immunity, thereby affecting susceptibility to viral pathogens. A recent *in vitro* study found that DMPA suppresses both innate and adaptive arms of immunity affecting host resistance to invading pathogens (18). Others also evaluated responsiveness of T cells by *ex vivo* phorbol myristate acetate and ionomycin stimulation of PBMCs isolated from women using DMPA and NET-EN (180 days), and found that the percentages of IFN-γ and TNF-α producing CD4 and CD8 T cells, as well as expression of immunosuppressive markers (CTLA-4 and PD-1), significantly decreased with DMPA but showed no significant change with NET-EN (22, 23, 56). A more recent study demonstrated that female sex workers using DMPA had increased genital inflammation, plasma IFN-γ and TNF-α levels, and increased CD4 T cells that linked to increased HIV-1 susceptibility (57). While several clinical, *in vitro* or *ex vivo* studies revealed that DMPA depletes inflammatory cytokine and chemokine production by T cells, dendritic cells or macrophages, many other studies showed DMPA increases cytokine and chemokine secretions by T cells and APCs (reviewed by Heffron et al.) (17). Interestingly, no such decreased cytokine or chemokine production by T cells or APCs was shown for NET-EN treatment, except in a few studies that reported NET-EN and DMPA show similar T cell and APC cytokine and chemokine responses (22, 23).

Given that available data showing DMPA affects immune responses are inconsistent or inconclusive, and definitive data for NET-EN are limited, the present study aimed to examine the frequencies and populations of CD4 and CD8 T cells in this model of OVX mice treated with DMPA or NET-EN. We first assessed the effects of NET-EN and DMPA on CD4 and CD8 T cell subset distribution in the vaginal tract and spleen to examine their effects at local or systemic levels. We found increased frequencies of CD8 T cells as well as IFN-γ+ CD4 and CD8 T cells both in VT and spleen with NET-EN. In contrast, DMPA markedly reduced percentages of CD4 T cells but enhanced frequencies of TNF-α producing CD4 and CD8 T cells in the spleen. Until our study, specific mouse data on CD4 and CD8 T cell populations in VT in the context of DMPA and NET-EN treatments has been lacking. However, in contrast to our results, a human clinical study showed DMPA increased the populations of CD3, CD8 and CD45+ T cells in the VT (58). Another human *ex vivo* study revealed an inhibition of T cell functions with DMPA but not NET-EN (20), which corroborates our *in vivo* findings. Similarly, Quispe Calla et al. found that MPA treated human DCs inhibited T cell proliferation *in vitro* (57). We have previously shown that DMPA treatments for four weeks increased HIV-1 target CD4+ T and CD14+ cells in humanized mice (5). The differences in results in the context of CD4 and CD8 T responses to DMPA or NET-EN between our study and other studies could possibly be due to species differences (mouse vs human) and/or due to differences in experimental settings (*in vivo* vs *ex vivo*/*in vitro*, VT lymphocytes vs PBMC).

We next examined the IFN-γ and TNF-α responses to PMA/ionomycin stimulation by CD4 and CD8 T cells isolated from NET-EN and DMPA treated mice. Our findings clearly delineate distinct functional phenotypes of CD4 and CD8 T cell responses following NET-EN and DMPA treatments in OVX mice. These include pronounced T cell IFN-γ response with NET-EN, contrasting DMPA that induced predominantly TNF-α response. These differences in T cell phenotypes were more pronounced in the VT than spleen. Of note, although we found greater IFN-γ responses both in CD4 and CD8 T cells in the spleen with NET-EN, TNF-α responses were inconclusive. When comparing the effects of DMPA and NET-EN on immune cells in the spleen and the vaginal tract, we observed that the effects are more pronounced in the vaginal tract. This is likely because the vaginal tract is a sex hormone responsive site compared to spleen. The progesterone receptors, which are the primary mechanism by which both DMPA and NET-EN exert their contraceptive effects, are expressed by all epithelial and stromal cells in the female reproductive tract (49, 59, 60). While some of the immune cells express progesterone receptors, the non-immune cells of the FRT may contribute to the unique effects on immune cells seen in the FRT (59). In addition to the progesterone receptor, although both DMPA and NET-EN are progestin-only contraceptives, they differ remarkably in their binding affinity to glucocorticoid receptor (GR) (reviewed in Hapgood et al. (13). DMPA possesses high affinity for GR but NET-EN has low or no affinity to GR. While GRs are ubiquitously expressed in all innate and adaptive immune cells, they are also expressed by the FRT epithelium (12, 14, 17). In addition, NET-EN has been shown to be converted to ethinyl estradiol, a potent estrogen that binds to and activates estrogen receptor (ER) and ER is extensively expressed by immune cells, as well as in FRT epithelium. Therefore, DMPA and NET-EN have more mechanisms to interact with both immune cells as well as non-immune cells in the vaginal tract to induce stronger immune responses. Spleen, on the other hand, has abundant immune cells but lacks non-immune cells which can respond to DMPA and NET-EN (18). This may contribute towards the more pronounced effects of progestin-based hormones in vaginal tract compared to spleen. Importantly, the increased IFN-γ responses in CD4 and CD8 T cells in the vaginal tract with NET-EN treatments correlated with the decreased susceptibility, less genital pathology and longer survival to HSV-2 infection. IFN-γ is known to be the most critical cytokine for clearance of HSV-2 (28, 30, 31).

On the other hand, TNF-α is well known as potent inflammatory cytokine, and excess production leads to tissue damage (61). DMPA induces enhanced levels of TNF-α production by CD4 and CD8 T cells in VT, which was correlated with HSV-2 susceptibility, genital pathology and increased mortality we have observed in DMPA treated mice, when compared to NET-EN treated mice. This is well supported by our previous *in vitro* findings that TNF-α was associated with disruption of genital and intestinal epithelial barrier function, thereby facilitating local viral dissemination to induce pathological lesions (62). The activity of the progestin-based contraceptives is mediated through their ability to bind the different hormone receptors. Different progestins bind not only to the progesterone receptor (PR) but also the other steroid receptors. The relative binding affinity of DMPA for PR is about 65-98% of progesterone (100%) while that of NET-EN is ~27-34% (13). Therefore, both DMPA and NET-EN exert progesterone-like effects. NET-EN and progesterone have low to no binding affinity to glucocorticoid receptor (GR), unlike DMPA which is known for its strong binding affinity for GR resulting in immunosuppressive activities (12, 14, 17). Neither progesterone nor DMPA bind to ER, while NET-EN has low estrogenic activity primarily because it can be aromatized to ethinyl estradiol, a potent estrogen that can bind ER. However, at the serum levels that NET-EN is present this activity is very low. Interestingly, the low estrogenic effects of NET-EN may be the reason why the vaginal epithelium of mice treated with NET-EN showed thicker epithelium compared to OVX and DMPA treated mice. Both NET-EN and DMPA have been shown to have hypoestrogenic effects, although DMPA is much more potent than NET-EN.

Given these differential biological activities, various studies have examined the effect of progestins and compared them to endogenous hormones in the context of STI infections. Our previous studies have examined the effect of DMPA, estrogen and progesterone on HSV-2 infection. When we compared changes between DMPA with progesterone, we found that while DMPA increased susceptibility to HSV-2 by 100-fold compared to normal mice in diestrus, progesterone increased susceptibility by 10-fold (51, 52). In contrast E2 treated and mice in estrus were not susceptible to HSV-2 (30, 43, 47, 55). The increased susceptibility seen in DMPA and progesterone has been shown to be due to multiple factors including thinning of epithelial lining in vaginal mucosa, increased permeability, decreased production of antibodies, reduced cell mediated cytotoxic activity and antigen presentation ability by antigen presenting cells (14, 16, 20, 63–65). The estradiol mediated protection is also due to multiple effects including thickening of vaginal epithelium, increased T cells production of IFN-γ and IL-17 and lowering vaginal pH (30, 43, 47, 55, 66).

While our current study was done in OVX mice given DMPA or NET-EN to avoid mixing of effects of endogenous hormones with exogenous ones, a recent study reported similar results to ours in regard to the effects of DMPA and NET-EN in a model with endogenous hormones. In this study, WT C57BL/6J mice in estrus did not show any morbidity or mortality, but NET-EN treated mice showed delayed genital pathology and mortality compared to DMPA treated mice (4). Enhanced IFN-γ anti-viral CD4 and CD8 T cell response by NET-EN versus enhanced TNF-α response by DMPA in local tissue that we found as key findings from this study may have important clinical implications in the context of susceptibility to viral STIs following NET-EN or DMPA exposures and needs to be investigated further in clinical settings.

It is well known that mucus levels in the female genital tract are influenced by the menstrual cycle, hormones and hormonal contraceptives (37, 38). Nevertheless, how and to what extent the progestin contraceptives DMPA and NET-EN regulate mucus levels in the FRT and the association of mucin with HSV-2 or other STIs has not been previously studied. In this study, we investigated the role of DMPA and NET-EN on mucin production in the vaginal tract because mucin acts as a physical barrier preventing virus attachment and entry into the vaginal tract epithelium, by trapping virus and removing virus during active mucus turnover (40, 67). A recent study demonstrated that synthetic mucin gel provides protection by binding with viruses and removing them from mucosal surfaces and efficiently reduces the HIV-1 and genital HSV-2 infectivity for various cell types (67). This is corroborated with our study where NET-EN treated mice that had higher levels of cell-associated mucin and decreased HSV-2 infection. In contrast, DMPA treated mice showed lower levels of Muc1 protein associated with higher level of HSV-2 infection. These results provide evidence that increased mucin, provided by the presence of NET-EN prevents HSV-2 virus attachment and infection in the vaginal epithelium.

There are several ideas as to how FRT mucus may play role in pathogen clearance. Mucin, in addition to preventing viruses from attaching to the vaginal epithelium, is a high molecular weight largely glycosylated protein molecule that can trap viruses and subsequently remove them from the vaginal tract, within vaginal discharge (34, 41). Mucin is known to be a crucial component of innate immunity by activating the TLR signaling pathway during airway infection caused by bacterial and viral pathogens (34–36). Although this phenomenon has not been evaluated in the FRT, it was shown that the interaction of Muc1 with TLR3 suppresses TNF-α release and thus limits the initial inflammatory response (36, 68). Future investigation is needed to dissect the underlying molecular mechanisms that may elucidate the link to DMPA and NET-EN induction of differential mucin production and HSV-2 inhibition. Notably, this is the first study that highlighted mucin as a possible regulating factor for HSV-2 infection in the context of DMPA and NET-EN exposure in mice. Future studies should focus on developing strategies to induce increased amounts of mucins in the FRT that could be a viable option to prevent or control HSV-2 and other STIs.

In addition to its effect on improving protection against pathogens by trapping them, mucin production by NET-EN could be a useful intervention for Genitourinary Syndrome of Menopause (GSM). GSM is a vulvovaginal atrophy that correlated with a variety of genitourinary symptoms and signs associated with menopause and is directly related to decreased circulating estrogen levels. Delineating clinical signs include vaginal dryness, pruritis, burning, irritation, dysuria and recurrent urinary infection that negatively impacts women’s quality of life. Approximately 1.2 billion women worldwide will be menopausal or postmenopausal by the year 2030 and half of the women will be affected by GSM (69). Therefore, development of appropriate treatment interventions is highly warranted. Current treatments include vaginal moisturizer, vaginal lubricants and vaginal estrogen therapies. OVX animal model including OVX ewe (70) and OVX mouse (71) model have proved effective in understanding GSM. Our findings that NET-EN induced increased mucin production in the vaginal tract of OVX mice that do not have any endogenous hormones, similar to menopausal women suggests that further studies to validate the estrogenic effects of NET-EN in enhancing mucin production could be vital as new treatment strategy for GSM in menopausal women.

Of note in this study, we have described the effects of different progestins on the changes seen in tissue resident T cells in the vaginal tract and similar changes seen systemically in spleen T cells in the absence of any antigen exposure (after 3 weeks of exposure to these hormones only) (**Figures 2**–**6**). We show that these changes may impact the outcome of primary exposure to HSV-2 (**Figures 1**, **7**). That sex hormones can alter T cell responses directly (transcriptional regulation) as well as indirectly affect T cell development and functions (through effects on thymic stromal cells and innate cells) is well established (72). Our results indicate that changes in the proportion and type of T cells in NET-EN treated mice may improve the primary immune response to HSV-2 infection. Because this was the first exposure to HSV-2, we did not examine the antigen specific responses, rather we would posit that the enriched proportion of IFN-γ producing T cells in the vaginal tract and spleen in response to NET-EN treatment would assist in rapid primary response to viral infection, since IFN-γ has been shown to be the main correlate of protection from HSV-2 (28–30). Previous studies have shown that in primary viral infections while some of the T cell expansion and activation is antigen specific, there is also bystander activation and/or cross-reactive stimulation of non-specific T cells which helps in viral clearance (73). The increased proportion of IFN-γ producing T cells in the vaginal tract induced by NET-EN may contribute to the delay in pathology and death.

Taken together, our results demonstrate that NET-EN-treated mice show delayed onset of genital pathology and delayed mortality from HSV-2 compared to with DMPA-treated mice. The anti-viral effects of NET-EN were associated with enhanced CD4 and CD8 T cell IFN-γ and mucin production in the vaginal tract compared to DMPA. We conclude from the above findings that NET-EN may be a more protective and safer contraceptive than DMPA in this pre-clinical model and can reduce the risks and impact of HSV-2 infection.

## Data Availability

The raw data supporting the conclusions of this article will be made available by the authors, without undue reservation.

## Supplementary material

Supplementary Figure 1 Serum levels of NET and MPA in OVX mice treated with NET-EN or DMPA. C57BL/6 mice were ovariectomized and 2 weeks later, NET-EN 2.5 mg and 5 mg pellets were inserted subcutaneously or 2 mg DMPA injected subcutaneously. Serum was collected 1 or 3 weeks after treatments. Serum was analyzed by high performance liquid chromatography paired with mass spectrometry. **(A)** Serum levels of NET were measured 1 and 3 weeks after treatment with either 2.5 mg or 5 mg of NET-EN or untreated OVX mice (UT). **(B)** Serum levels of MPA were measured 1 and 3 weeks after DMPA treatment or untreated OVX mice (UT). Bars indicate mean ± SEM for n= 3 mice in each group.

Supplementary Figure 2 Experimental design for examining the effects of NET-EN and DMPA treatments on immune cells phenotypes and functions and mucin production. C57BL/6 mice were ovariectomized, and 2 weeks later, NET-EN (2.5 mg) pellets were inserted subcutaneously at the scruff region of a group of mice (n=5-10), or 2 mg DMPA was injected subcutaneously in another group of mice (n=5-10), while untreated control mice received no hormonal contraceptive (n=5-10). Vaginal washes were collected at week 1, 2 and 3 after contraceptive treatments to determine the secreted mucin levels by ELISA. Mice were sacrificed after 3 weeks of NET-EN and DMPA treatments, vaginal tract tissues and spleen were harvested, single cells isolated and subjected to flow cytometry analyses for immune cells populations and their cytokine production (IFN-γ and TNF-α). In a separate set of similar experiments, vaginal washes were collected at week 1, 2 and 3 after NET-EN and DMPA treatments or untreated mice to assess mucin levels by ELISA. Mice were then euthanized, vaginal tissue sections harvested and subjected to PAS staining and Muc1 immunohistochemistry to determine mucin production in the vaginal tract.

Supplementary Figure 3 Gating strategy for Flow cytometry data analyses of vaginal tissue T cells. Isolated vaginal tract cells were gated: **(A)** to identify mononuclear lymphocyte populations; **(B)** for single cells; **(C)** viable cells; (D) CD3+ T cells, and then **(E)** CD4+ and CD8+ cells were identified. With IFN-γ staining, CD4+ T cells were further gated based on **(F)** FMO for IFN-γ to show positive fluorescence rectangle **(G)** IFN-γ+ CD4 T cells; **(H)** FMO for TNF-α to show positive fluorescence rectangle and **(I)** TNF-α + CD4 T cells. Similar gating approaches were applied for analyzing IFN-γ+ or TNF-α+ CD8 T cells (not shown).

Supplementary Figure 4 Gating strategy for Flow cytometry data analyses of spleen T cells. Isolated spleen cells were gated: **(A)** to identify mononuclear lymphocyte populations; **(B)** for single cells; **(C)** viable cells; **(D)** CD3+ T cells, and then **(E)** CD4+ and CD8+ cells were identified. With IFN-γ staining, CD4+ T cells were further gated based on **(F)** FMO for IFN-γ to show positive fluorescence rectangle **(G)** IFN-γ+ CD4 T cells; **(H)** FMO for TNF-α to show positive fluorescence rectangle and **(I)** TNF-α + CD4 T cells. Similar gating approaches were applied for analyzing IFN-γ+ or TNF-α+ CD8 T cells (not shown).

Supplementary Figure 5 Comparative analyses of mucin production in the vaginal tract of OVX mice treated with NET-EN, DMPA or left untreated without or with HSV-2 infection. PAS and Muc1 staining intensities for images of vaginal tissues for different hormonal treatments showed in **Figure 6C** and in **Figure 7A** were quantified using Fiji ImageJ software as depicted in bar graphs. Bars for hormonal treatments groups (no HSV-2 infection, **Figure 6C**) indicate mean ± SEM for n=9 samples (3 images per mouse vaginal tissue, n=3 mice per treatment) and bars for hormones+HSV-2 groups (**Figure 7A**) indicate mean ± SEM for n=10 images (n=5 mice per treatment and 2 images per vaginal tissue). All data are drawn from 2 independent experiments and analyzed utilizing the one-way ANOVA with Tukey’s multiple comparison test; P <0.05 considered as significant. Comparisons made between same hormone treatments only.
