# Supplementary figures and images for: NET-EN treatment leads to delayed HSV-2 infection, enhanced mucin and T cell functions in the female genital tract when compared to DMPA in a preclinical mouse model
Source: Front Immunol. 2024 Nov 6;15:1427842. doi: 10.3389/fimmu.2024.1427842 (PMC11576457; doi:10.3389/fimmu.2024.1427842)

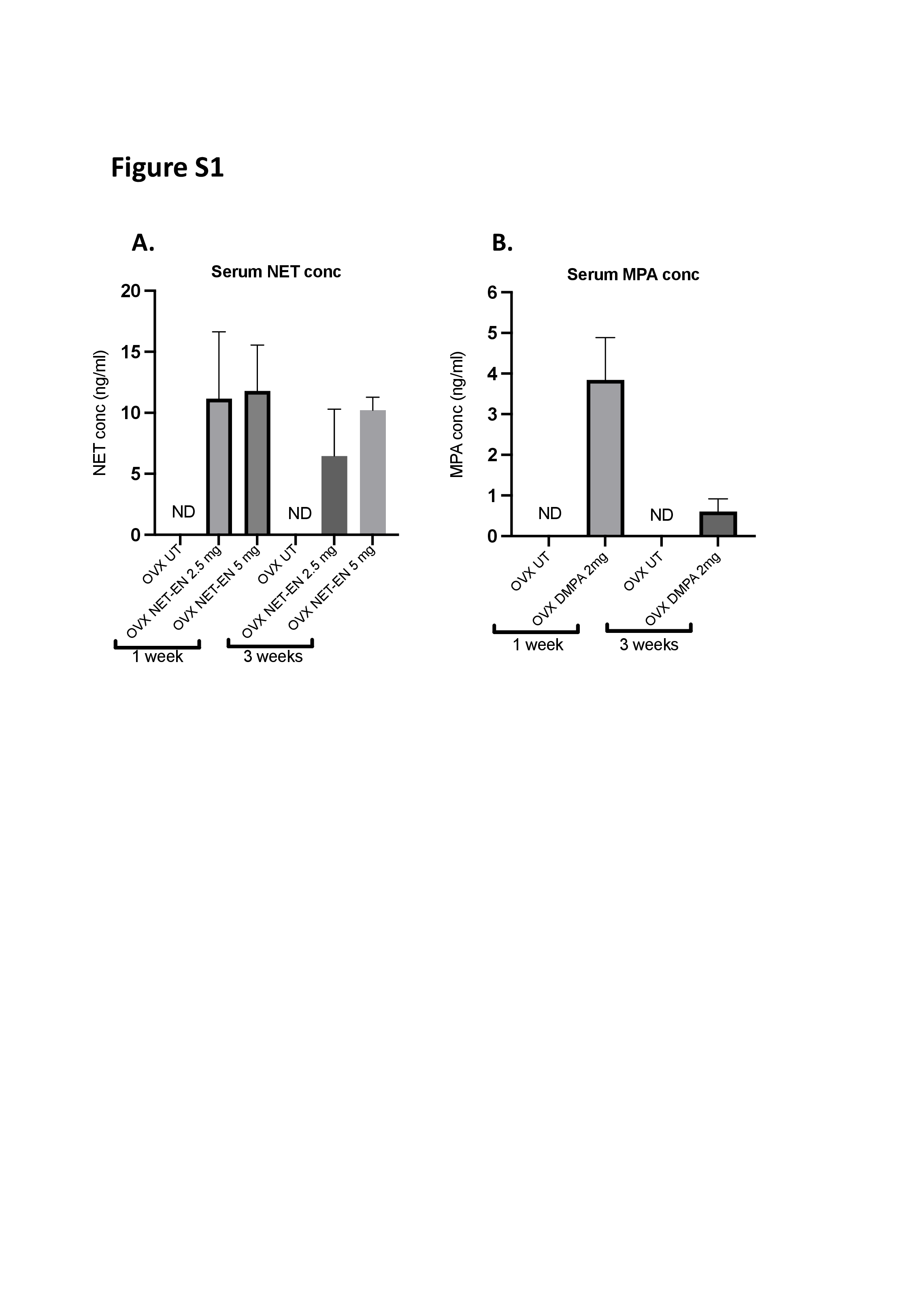

Supplement: Supplementary Figure 1 — Serum levels of NET and MPA in OVX mice treated with NET-EN or DMPA. C57BL/6 mice were ovariectomized and 2 weeks later, NET-EN 2.5 mg and 5 mg pellets were inserted subcutaneously or 2 mg DMPA injected subcutaneously. Serum was collected 1 or 3 weeks after treatments. Serum was analyzed by high performance liquid chromatography paired with mass spectrometry. (A) Serum levels of NET were measured 1 and 3 weeks after treatment with either 2.5 mg or 5 mg of NET-EN or untreated OVX mice (UT). (B) Serum levels of MPA were measured 1 and 3 weeks after DMPA treatment or untreated OVX mice (UT). Bars indicate mean ± SEM for n= 3 mice in each group. [file Image1.tiff]

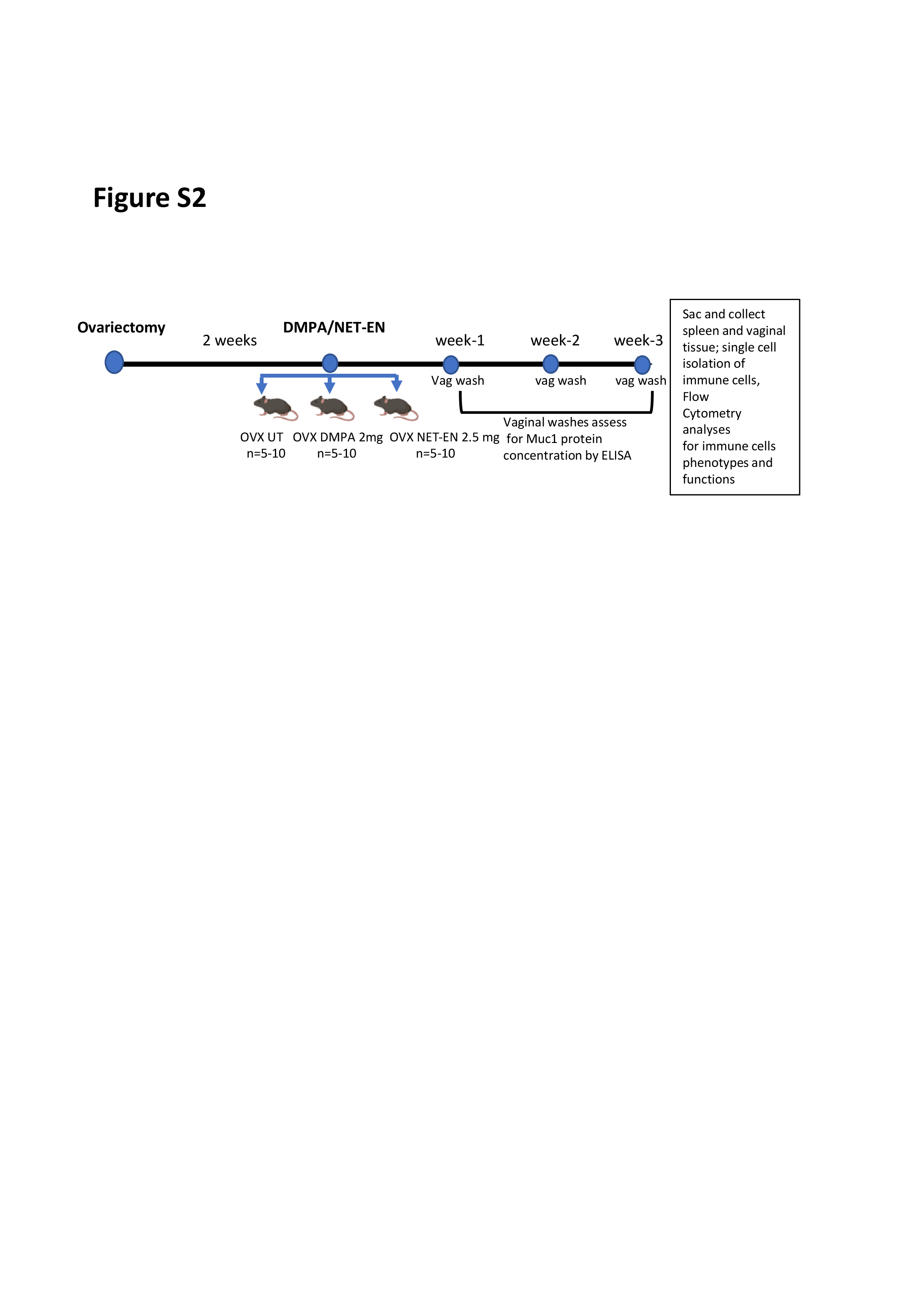

Supplement: Supplementary Figure 2 — Experimental design for examining the effects of NET-EN and DMPA treatments on immune cells phenotypes and functions and mucin production. C57BL/6 mice were ovariectomized, and 2 weeks later, NET-EN (2.5 mg) pellets were inserted subcutaneously at the scruff region of a group of mice (n=5-10), or 2 mg DMPA was injected subcutaneously in another group of mice (n=5-10), while untreated control mice received no hormonal contraceptive (n=5-10). Vaginal washes were collected at week 1, 2 and 3 after contraceptive treatments to determine the secreted mucin levels by ELISA. Mice were sacrificed after 3 weeks of NET-EN and DMPA treatments, vaginal tract tissues and spleen were harvested, single cells isolated and subjected to flow cytometry analyses for immune cells populations and their cytokine production (IFN-γ and TNF-α). In a separate set of similar experiments, vaginal washes were collected at week 1, 2 and 3 after NET-EN and DMPA treatments or untreated mice to assess mucin levels by ELISA. Mice were then euthanized, vaginal tissue sections harvested and subjected to PAS staining and Muc1 immunohistochemistry to determine mucin production in the vaginal tract. [file Image2.tiff]

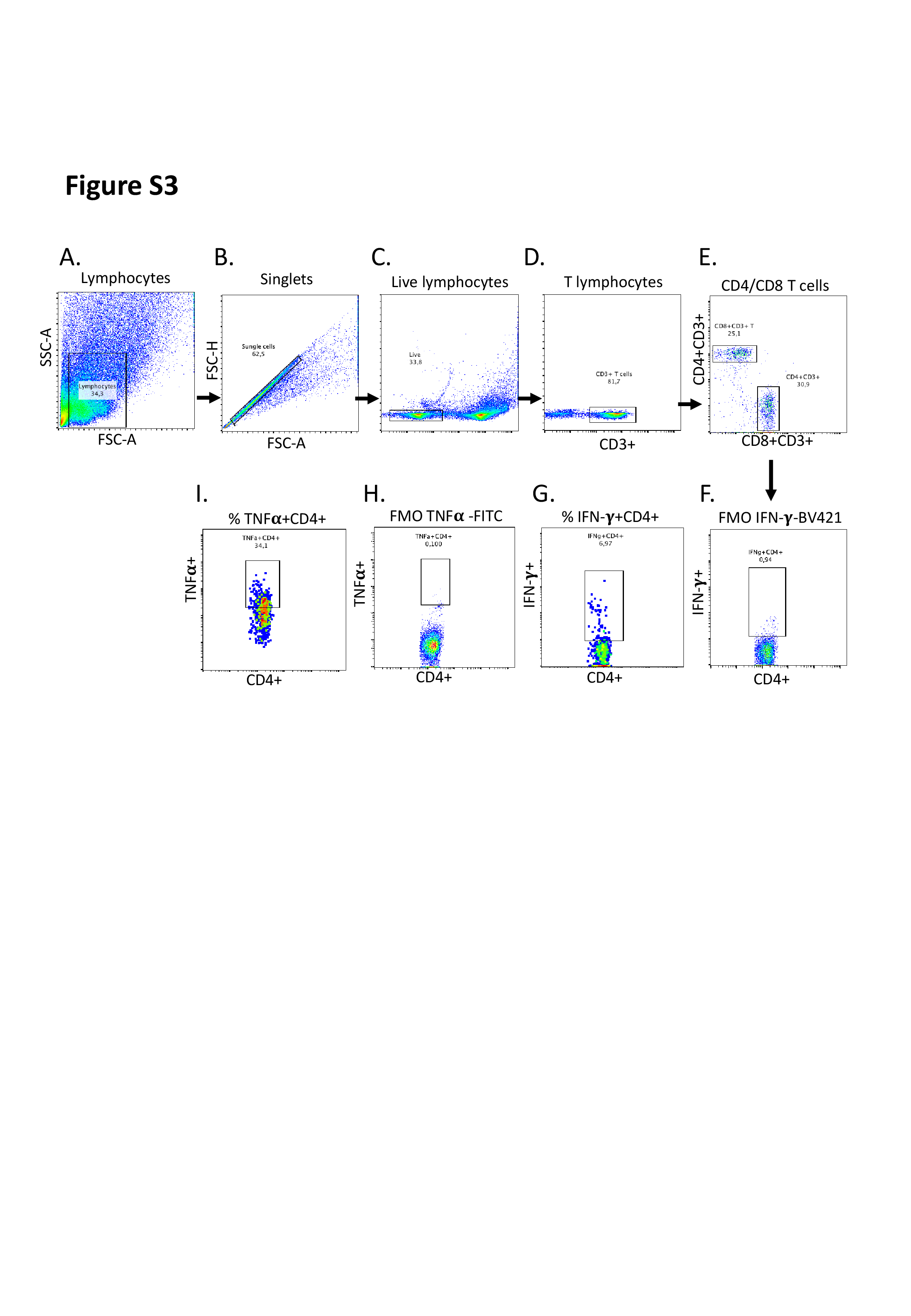

Supplement: Supplementary Figure 3 — Gating strategy for Flow cytometry data analyses of vaginal tissue T cells. Isolated vaginal tract cells were gated: (A) to identify mononuclear lymphocyte populations; (B) for single cells; (C) viable cells; (D) CD3+ T cells, and then (E) CD4+ and CD8+ cells were identified. With IFN-γ staining, CD4+ T cells were further gated based on (F) FMO for IFN-γ to show positive fluorescence rectangle (G) IFN-γ+ CD4 T cells; (H) FMO for TNF-α to show positive fluorescence rectangle and (I) TNF-α + CD4 T cells. Similar gating approaches were applied for analyzing IFN-γ+ or TNF-α+ CD8 T cells (not shown). [file Image3.tiff]

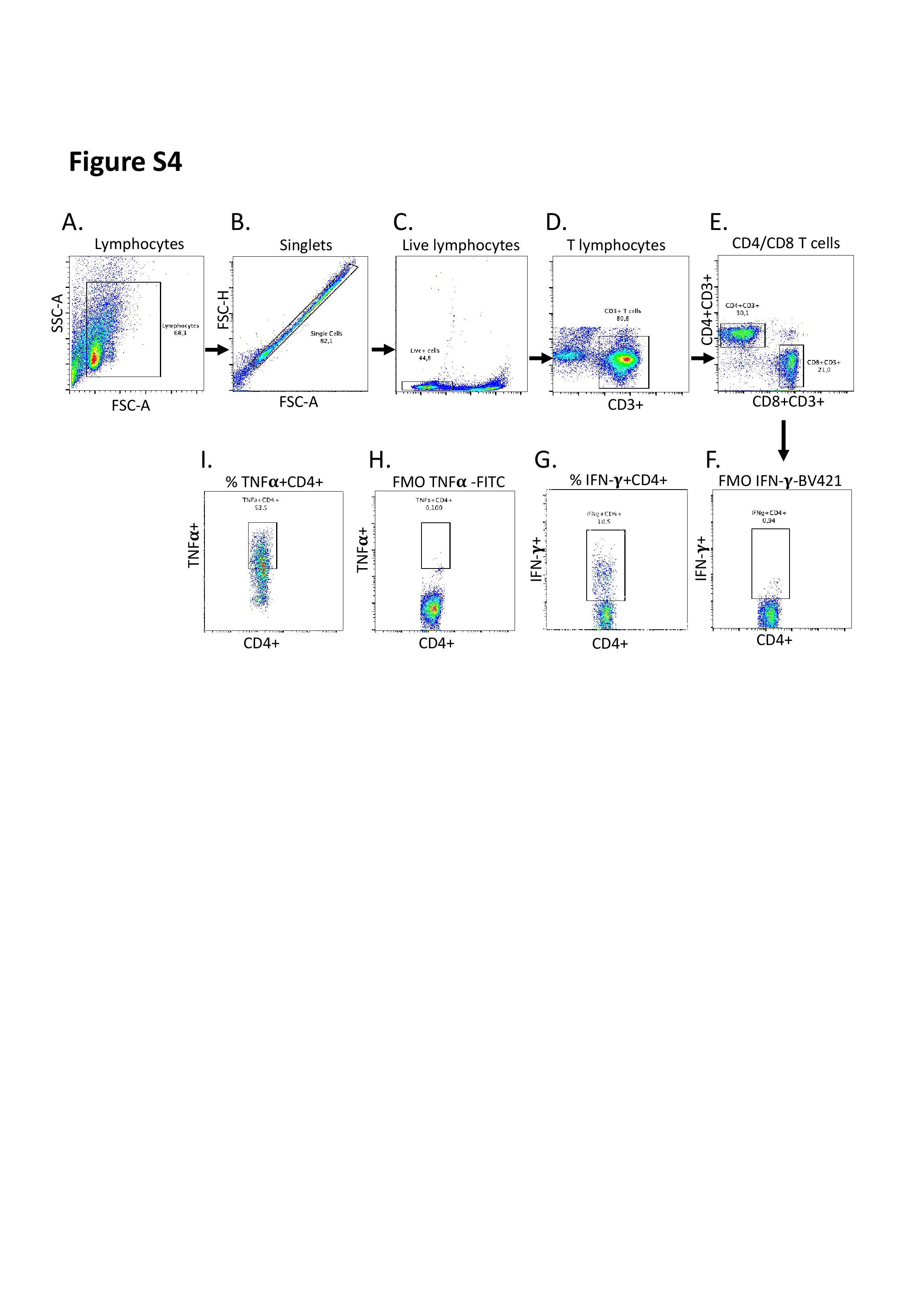

Supplement: Supplementary Figure 4 — Gating strategy for Flow cytometry data analyses of spleen T cells. Isolated spleen cells were gated: (A) to identify mononuclear lymphocyte populations; (B) for single cells; (C) viable cells; (D) CD3+ T cells, and then (E) CD4+ and CD8+ cells were identified. With IFN-γ staining, CD4+ T cells were further gated based on (F) FMO for IFN-γ to show positive fluorescence rectangle (G) IFN-γ+ CD4 T cells; (H) FMO for TNF-α to show positive fluorescence rectangle and (I) TNF-α + CD4 T cells. Similar gating approaches were applied for analyzing IFN-γ+ or TNF-α+ CD8 T cells (not shown). [file Image4.tiff]

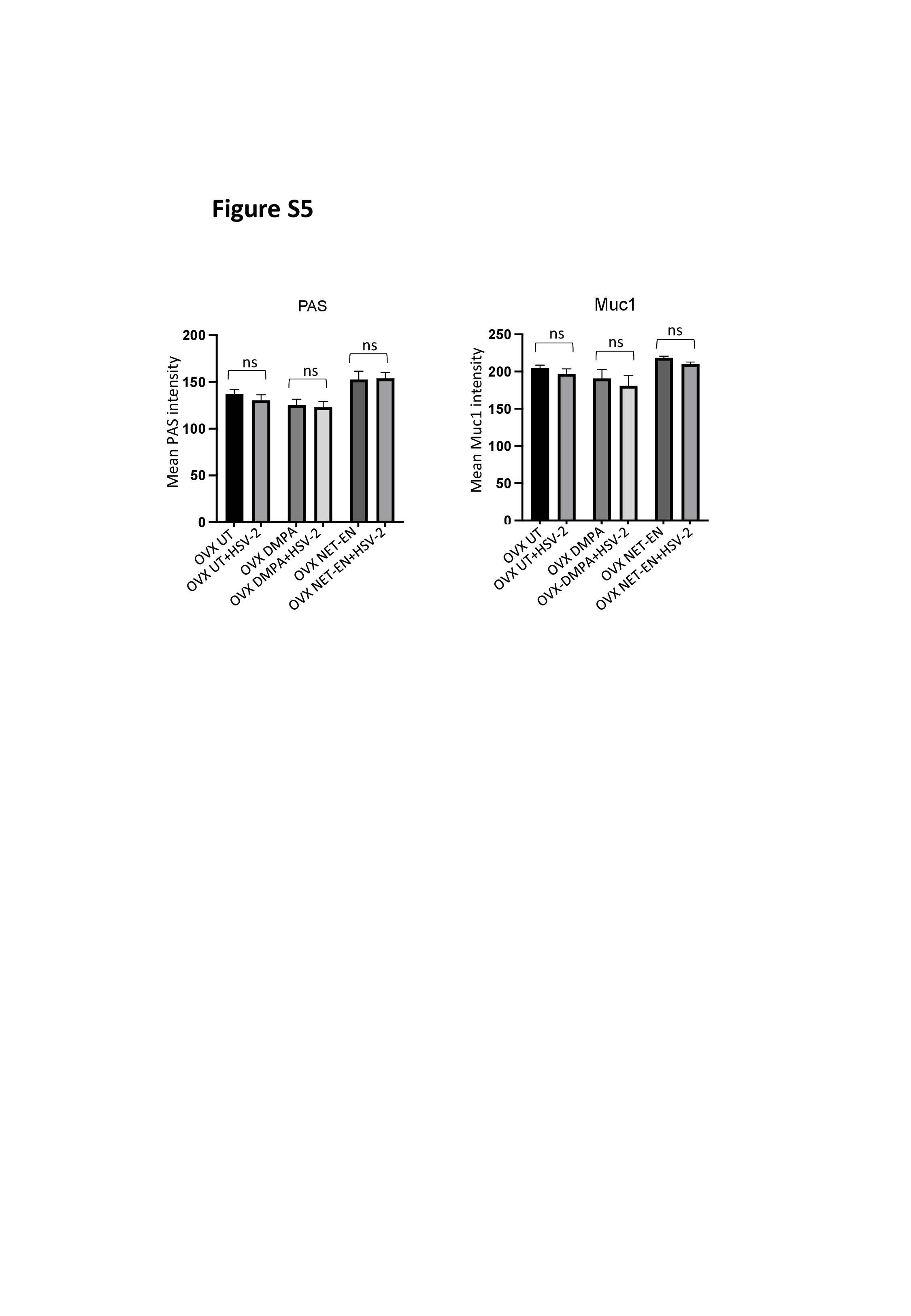

Supplement: Supplementary Figure 5 — Comparative analyses of mucin production in the vaginal tract of OVX mice treated with NET-EN, DMPA or left untreated without or with HSV-2 infection. PAS and Muc1 staining intensities for images of vaginal tissues for different hormonal treatments showed in Figure 6C and in Figure 7A were quantified using Fiji ImageJ software as depicted in bar graphs. Bars for hormonal treatments groups (no HSV-2 infection, Figure 6C ) indicate mean ± SEM for n=9 samples (3 images per mouse vaginal tissue, n=3 mice per treatment) and bars for hormones+HSV-2 groups ( Figure 7A ) indicate mean ± SEM for n=10 images (n=5 mice per treatment and 2 images per vaginal tissue). All data are drawn from 2 independent experiments and analyzed utilizing the one-way ANOVA with Tukey’s multiple comparison test; P <0.05 considered as significant. Comparisons made between same hormone treatments only. [file Image5.tiff]
